# ERR**γ** suppression by Sirt6 alleviates cholestatic liver injury and fibrosis

**DOI:** 10.1172/jci.insight.137566

**Published:** 2020-09-03

**Authors:** Lihua Hao, In Hyuk Bang, Jie Wang, Yuancheng Mao, Jae Do Yang, Soon-Young Na, Jeong Kon Seo, Hueng-Sik Choi, Eun Ju Bae, Byung-Hyun Park

**Affiliations:** 1 Department of Biochemistry and; 2 Department of Surgery, Chonbuk National University Medical School, Jeonju, Jeonbuk, South Korea.; 3 School of Biological Sciences and Technology, Chonnam National University, Gwangju, South Korea.; 4 UNIST Central Research Facilities, UNIST, Ulsan, South Korea.; 5 College of Pharmacy, Chonbuk National University, Jeonju, Jeonbuk, South Korea.

**Keywords:** Hepatology, Metabolism, Fibrosis

## Abstract

Orphan nuclear receptor estrogen-related receptor γ (ERRγ) stimulates bile acid production; however, the role and the regulatory mechanism of ERRγ in cholestatic liver disease are largely unknown. This study identifies that Sirt6 is a deacetylase of ERRγ and suggests a potentially novel mechanism by which Sirt6 activation alleviates cholestatic liver damage and fibrosis through regulating ERRγ. We observed that hepatic expression of Sirt6 is repressed, whereas hepatic expression of ERRγ is upregulated in murine cholestasis models. Hepatocyte-specific Sirt6-KO mice were more severely injured after a bile duct ligation (BDL) than WT mice, and adenoviral reexpression of Sirt6 reversed liver damage and fibrosis as demonstrated by biochemical and histological analyses. Mechanistically, Sirt6 deacetylated ERRγ, thereby destabilizing ERRγ and inhibiting its transcriptional activity. Elimination of hepatic ERRγ using Ad-shERRγ abolished the deleterious effects of Sirt6 deficiency, whereas ERRγ overexpression aggravated cholestatic liver injury. Administration of a Sirt6 deacetylase activator prevented BDL-induced liver damage and fibrosis. In patients with cholestasis, Sirt6 expression was decreased, whereas total ERRγ and acetylated ERRγ levels were increased, confirming negative regulation of ERRγ by Sirt6. Thus, Sirt6 activation represents a potentially novel therapeutic strategy for treating cholestatic liver injury.

## Abstract 2

Sirt6 activation alleviates cholestatic liver damage and fibrosis in murine cholestasis models by deacetylation of ERRγ.

## Graphical abstract

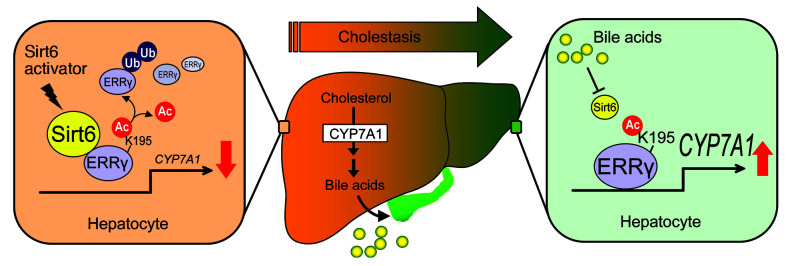

## Introduction

Bile acids (BAs) are formed in the liver from cholesterol and secreted into the intestine, where they facilitate fat digestion and absorption. BAs also affect glucose and lipid homeostasis by activating nuclear receptors and cell signaling pathways. Impaired flow or enhanced formation of BAs induces liver damage, which is referred to as cholestatic liver injury (1, 2). Although the detailed mechanism by which cholestasis induces hepatic and biliary damage remains unclear, the impact of cholestasis varies from simple injury and inflammation to fibrosis/cirrhosis and liver failure (2). Current medications for treating cholestasis are limited, and thus the identification of potentially novel therapeutic targets for preventing or treating cholestasis is important. Ursodeoxycholic acid is the first BA-based treatment; this drug exerts an anticholestatic effect mainly by stimulating BA secretion (3). Recently, obeticholic acid as a farnesoid X receptor (FXR) agonist has been approved and used for the treatment of primary biliary sclerosis (4).

Under physiological conditions, BA synthesis is tightly regulated by a negative feedback mechanism mediated by nuclear BA receptor FXR (5, 6). BA activates FXR, which directly induces the expression of multiple target genes, including small heterodimer partner (SHP) and fibroblast growth factor 19 in the liver and intestine, respectively. In turn, SHP inhibits BA synthesis via repression of cholesterol 7α-hydroxylase (CYP7A1), a rate-limiting enzyme involved in BA synthesis, as well as other enzymes involved in BA formation. *CYP7A1* has a binding site for orphan nuclear receptor estrogen-related receptor γ (ERRγ) in its promoter region (7). Thus, ERRγ can induce the expression of *CYP7A1*, and this is markedly inhibited by SHP. Of the 3 ERR subtypes (α, β, and γ), only ERRγ specifically promotes *CYP7A1* transcription. ERRγ is constitutively active in the absence of the cognate ligand (8), and an inverse agonist that inhibits its activity after binding to ERRγ has been shown to have a therapeutic effect in many metabolic diseases and cancer (9). ERRγ undergoes various posttranslational modifications, including phosphorylation, sumolyation, and O-GlcNAcylation, all of which alter its transcriptional activity (9). Despite the important effect of posttranslational modification on ERRγ function, the potential impact of (de)acetylation of ERRγ has not been studied.

Sirtuins (Sirt1–Sirt7) are NAD^+^-dependent histone III deacetylases. The role of Sirt6 in chromatin remodeling, cell proliferation/differentiation, and metabolism has been studied extensively and is mediated by its ability to deacetylate histones at lysine residues (H3K9, H3K18, and H3K56) as well as nonhistone substrates (10, 11). We and others previously reported that hepatic deletion of Sirt6 promoted alcoholic and nonalcoholic fatty liver disease (NAFLD), nonalcoholic steatohepatitis (NASH), and fibrosis (10, 12–14). BA level is increased in the livers of patients with NASH, and synthesis and serum levels of BAs are correlated with the disease severity of NAFLD (15, 16). However, the role of Sirt6 in BA synthesis and homeostasis is unclear.

Given the nuclear retention of Sirt6 and its deacetylase activity, we hypothesized that Sirt6 may deacetylate ERRγ, thereby modifying its function. The aims of the present study were as follows: (a) evaluate if deficiency or reexpression of Sirt6 in liver affects obstructive cholestatic liver injury, (b) investigate if ERRγ is a deacetylation target of Sirt6 and if ERRγ mediates the deleterious effect of Sirt6 deficiency, and (c) evaluate if a Sirt6 activator has a therapeutic effect against cholestatic liver injury. The findings of this study provide a proof of concept that activation of Sirt6 deacetylase is a potential therapeutic option for treatment of cholestatic liver injury through inhibition of ERRγ.

## Results

### Hepatic expression of Sirt6 is repressed, whereas hepatic expression of ERRγ is upregulated in murine cholestasis models.

**Figure 1:**
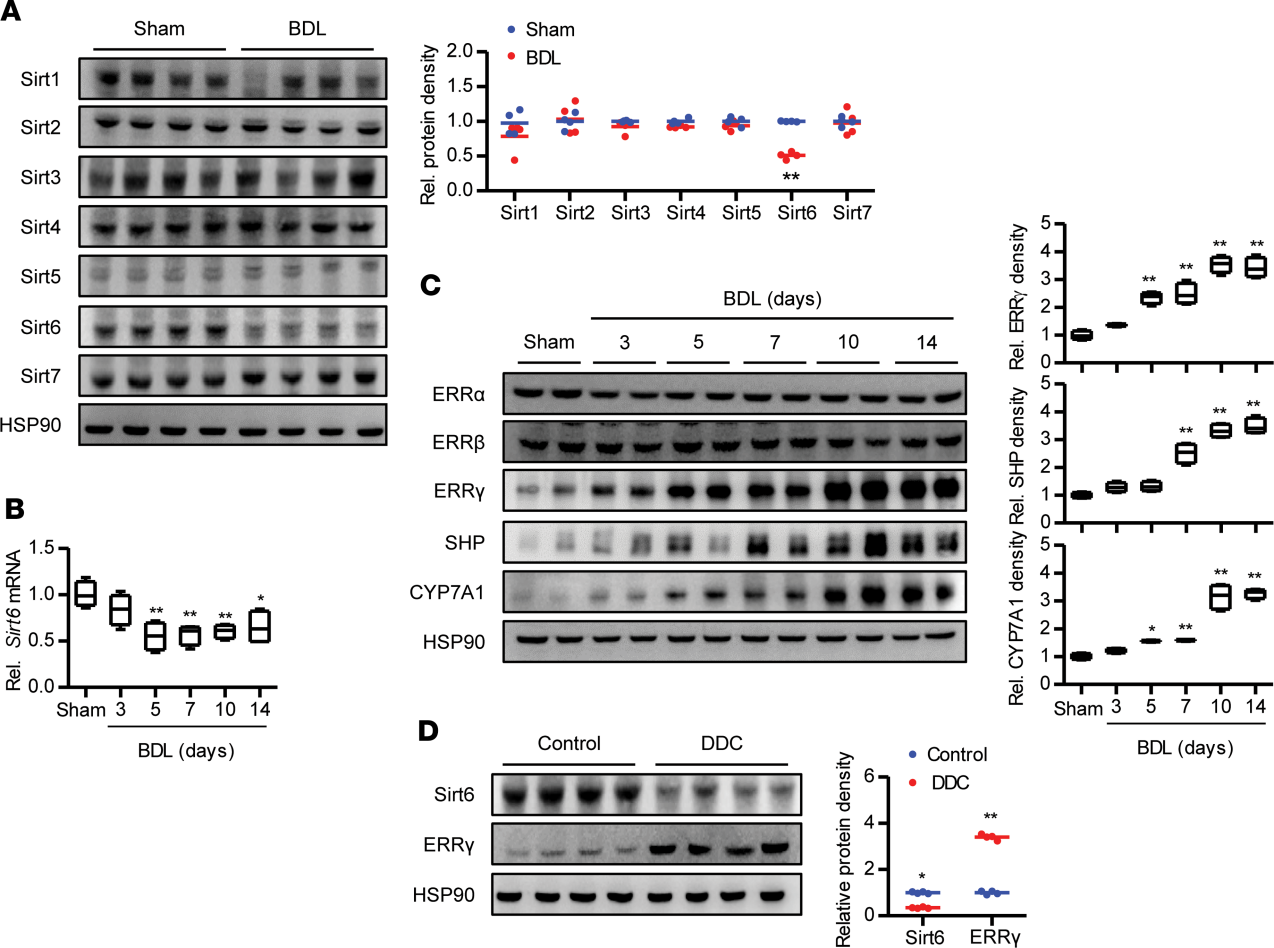
Hepatic expression of Sirt6 and ERRγ after experimental BDL or DDC feeding in mice. (**A**) Lysates were prepared from primary hepatocytes 10 days after BDL and sirtuin expression was determined by Western blot (*n* = 4). (**B** and **C**) After BDL, liver homogenates were subjected to quantitative PCR (*n* = 4) or Western blotting (*n* = 4). (**D**) Protein levels of Sirt6 and ERRγ were analyzed in mice fed a DDC diet (*n* = 4). Values are shown as mean ± SEM. Comparisons were made using paired 2-tailed Student’s *t* test (**A** and **D**) or 1-way ANOVA followed by Tukey’s multiple-comparisons test (**B** and **C**). *P < 0.05 and **P < 0.01 versus sham or control mice.

We first determined the expression of sirtuin members in hepatocytes isolated from bile duct ligation (BDL) mice. Both protein and mRNA levels of Sirt6 were markedly reduced in the hepatocytes of BDL mice compared with those that underwent a sham operation, with no change in levels of other sirtuins (Figure 1, A and B). Treatment of hepatocytes with different BAs, i.e., taurocholic acid, chenodeoxycholic acid, or deoxycholic acid, also resulted in a decrease in Sirt6 expression (Supplemental Figure 1; supplemental material available online with this article; https://doi.org/10.1172/jci.insight.137566DS1), indicating that BA signaling in hepatocytes directly inhibits Sirt6 expression. Interestingly, BA treatment of hepatocytes also increased the expression of ERRγ protein. When we evaluated the expression of ERR subtypes, ERRγ protein expression was noticeably and persistently increased in liver after BDL without alteration in ERRα or ERRβ expression (Figure 1C). Although BDL operation induced a significant increase in SHP expression, CYP7A1 expression was not decreased but significantly increased, likely driven by ERRγ (Figure 1C). Opposite changes in Sirt6 and ERRγ protein levels were also demonstrated in cholestatic liver samples from mice that were fed a diet supplemented with 0.1% 3,5-diethoxycarbonyl-1,4-dihydrocollidine (DDC) (Figure 1D). Overall, our results indicate that BA treatment or overloading represses Sirt6 expression and that Sirt6 reduction may be involved in upregulation of ERRγ and CYP7A1.

### BDL-induced liver injury is aggravated by Sirt6 deficiency.

**Figure 2:**
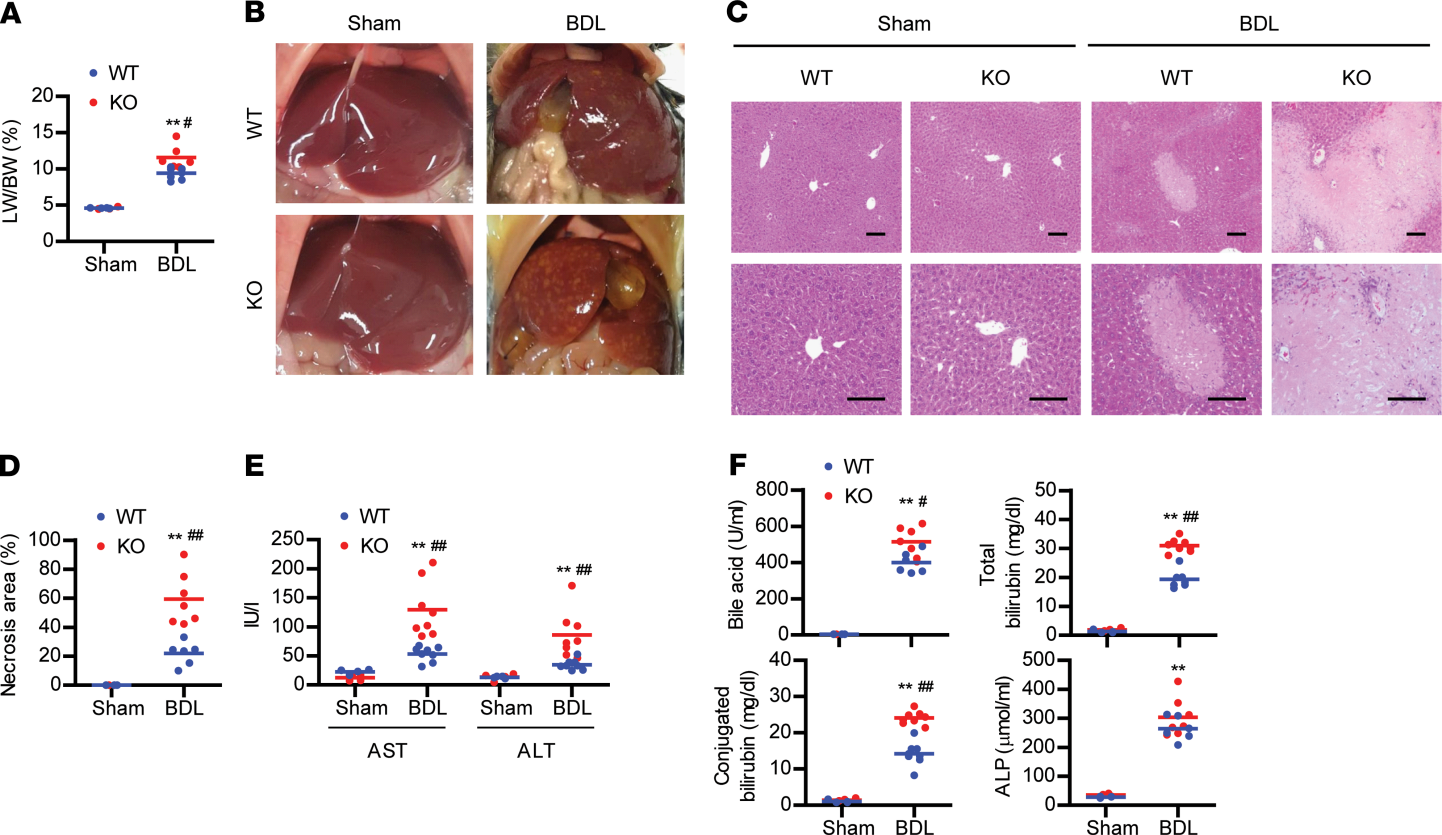
Aggravation of BDL-induced liver injury by Sirt6 deficiency. (**A** and **B**) Liver weight/body weight and photographs of representative mouse liver were obtained 10 days after BDL (*n* = 4–8). (**C** and **D**) Liver necrosis was assessed by H&E staining. Scale bar: 200 μm. The area of necrosis was measured (*n* = 3–6). (**E** and **F**) Serum levels of AST, ALT, bile acid, total bilirubin, conjugated bilirubin, and ALP were analyzed (*n* = 4–8). Values are shown as mean ± SEM. Comparisons were made using paired 2-tailed Student’s *t* test. **P < 0.01 versus WT sham; ^#^*P* < 0.05 and ^##^*P* < 0.01 versus WT BDL.

To directly determine the relevance of Sirt6 reduction in cholestatic liver injury, we performed BDL in hepatocyte-specific Sirt6-KO (*Sirt6*^fl/fl^ *albumin-Cre*) mice and WT littermates. Results showed that BDL increased liver/body weight ratio by about 2-fold in WT mice and 2.5-fold in Sirt6-KO mice (Figure 2A). Gross examination and H&E staining of the liver clearly indicated severe and diffuse necrosis in KO mice (Figure 2, B–D). The elevation of serum levels of aspartate aminotransferase (AST), alanine aminotransferase (ALT), BAs, and bilirubin by BDL was significantly higher in KO mice than WT mice (Figure 2, E and F).

We further employed a well-established xenobiotic-induced cholangiopathy model; mice were fed a diet supplemented with 0.1% DDC for 1 week (17). Liver injury, inflammation, biliary fibrosis, and ductular reactions were aggravated in Sirt6-KO mice compared with WT mice (Supplemental Figure 2, A–C). In accordance with Sirt6 repression by BAs, DDC feeding also decreased Sirt6 expression but increased ERRγ expression, confirming opposite regulation of Sirt6 and ERRγ (Supplemental Figure 2D).

After BDL, circulating neutrophils acutely infiltrate liver tissue and are the predominant mediators of acute liver injury (18–20). Increased infiltration of neutrophils and macrophages in the liver of Sirt6-KO mice was confirmed by histopathological analyses; numbers of Gr-1– and F4/80–positive cells were significantly higher in Sirt6-KO mice than in WT liver (Supplemental Figure 3, A and B). TUNEL staining and Western blot analysis of Bax, cleaved caspase-3, and Bcl-2 suggested greater apoptotic cell death in the livers of KO mice than those of WT mice (Supplemental Figure 3, A–C). Liver fibrosis and intrahepatic bile duct reaction, measured by Sirius red staining, Western blots of α–smooth muscle actin (α-SMA) and collagen 1, and cytokeratin 19 (CK19) immunostaining, were significantly increased by BDL to a greater extent in KO mice than WT mice (Supplemental Figure 3, A–C). Consistently, mRNA levels of proinflammatory mediators, chemokines, and fibrosis-related genes were increased in KO mice (Supplemental Figure 3, D and E), and this was accompanied by the activation of ERK, JNK, and JAK2/STAT3 signaling pathways (Supplemental Figure 3C).

Cholestatic hepatocytes initiate inflammation by secreting proinflammatory cytokines and chemokines (20). To test the ability of hepatocytes to trigger inflammatory gene expression upon BA exposure, we employed coculture of hepatocytes and Kupffer cells (KCs). Hepatocytes from Sirt6-KO mice expressed greater amounts of *Tnfa*, *Il6*, *Cxcl1*, and *Cxcl2* in response to taurocholic acid exposure, and the level of these transcripts was even higher when hepatocytes were cocultured with KCs, whereas minimal induction of these genes was observed in WT hepatocytes cultured alone or with KCs (Supplemental Figure 4).

### Reexpression of Sirt6 in Sirt6-KO mice reverses BDL-induced liver injury.

**Figure 3:**
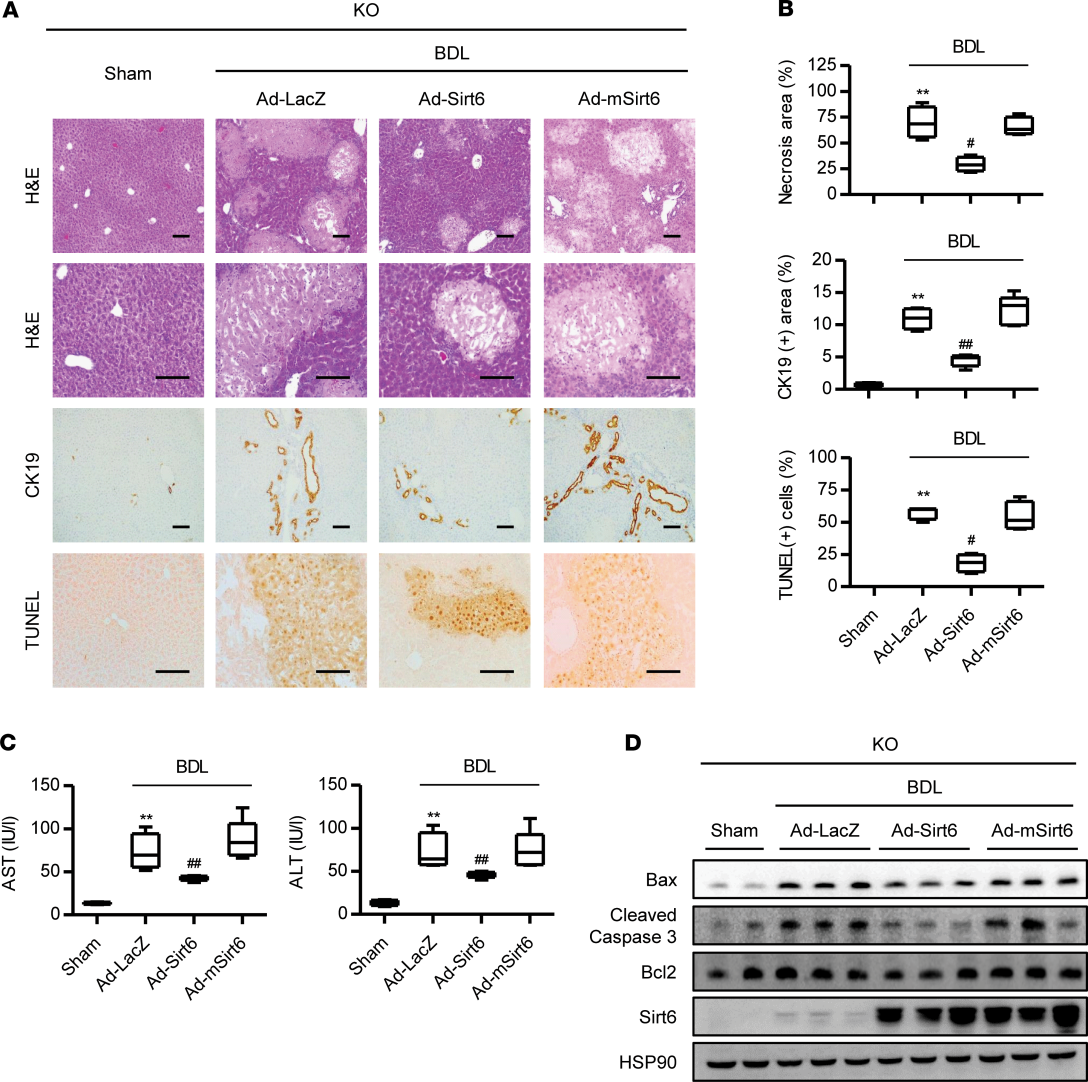
Attenuation of liver injury by reexpression of Sirt6 in BDL KO mice. Sirt6-KO mice were injected intravenously with 1 × 10^9^ PFU of Ad-LacZ, Ad-Sirt6, or Ad-mSirt6 and then subjected to BDL for 10 days. (**A**) Representative microscopic pictures of liver sections. Scale bar: 200 μm. (**B**) Necrosis (H&E), ductular reaction (cytokeratin 19), and apoptosis (TUNEL) were quantified (*n* = 4). (**C**) Serum levels of AST and ALT were measured (*n* = 5). (**D**) The protein levels involved in apoptosis-related pathways were analyzed (*n* = 3). Values are shown as mean ± SEM. Comparisons were made using 1-way ANOVA followed by Tukey’s multiple-comparisons test. **P < 0.01 versus sham; ^#^*P* < 0.05 and ^##^*P* < 0.01 versus Ad-LacZ BDL.

We next evaluated if BDL-induced liver injury in Sirt6-KO mice was restored by reexpression of Sirt6. Severe liver necrosis, intrahepatic bile ductular reaction, and apoptosis with increases in serum levels of AST and ALT in Sirt6-KO were significantly ameliorated by Ad-Sirt6 injection (Figure 3, A–C), whereas liver/body weight ratio did not change (Supplemental Figure 5A). Overexpression of deacetylase-mutant Sirt6 (Ad-mSirt6) did not result in an improvement in liver histology and injury markers, suggesting that the deacetylase activity of Sirt6 is required for the protective function of Sirt6 against cholestasis. Ad-Sirt6 consistently decreased the BDL-induction of Bax and cleaved caspase-3 and the increase in serum levels of BAs and bilirubin (Figure 3D and Supplemental Figure 5B).

### Sirt6 interacts physically with ERRγ to deacetylate this protein.

**Figure 4:**
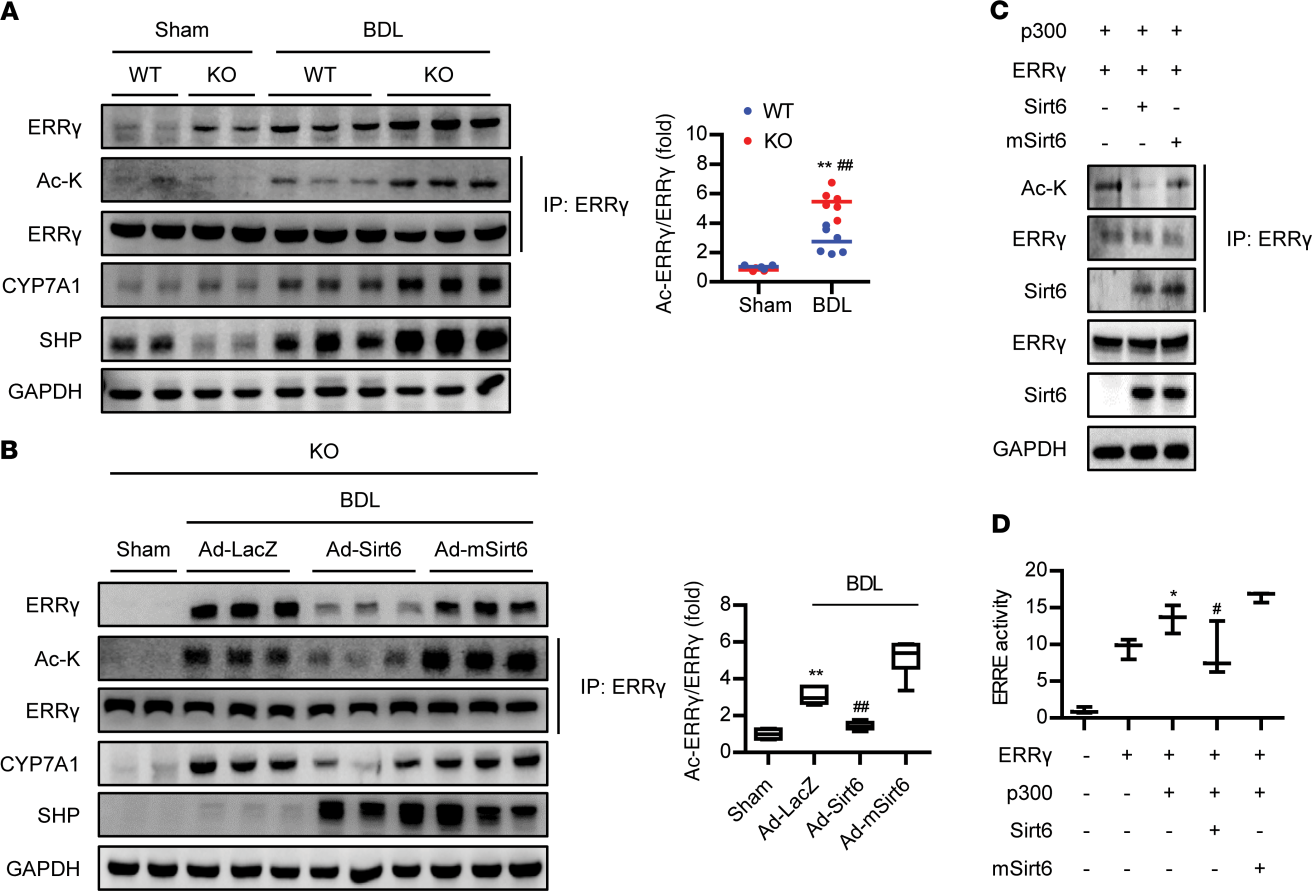
Deacetylation of ERRγ by Sirt6. (**A**) Protein levels of total ERRγ and acetylated ERRγ, CYP7A1, and SHP in WT and KO livers were examined by Western blotting (*n* = 6). (**B**) Sirt6-KO mice were injected intravenously with 1 × 10^9^ PFU of Ad-LacZ, Ad-Sirt6, or Ad-mSirt6 and then subjected to BDL. Acetylation of ERRγ was determined (*n* = 6). (**C**) After transfection of HEK293T cells, acetylation of ERRγ was determined. (**D**) ERRE-luciferase activity was determined (*n* = 5). Values are shown as mean ± SEM. Comparisons were made using paired 2-tailed Student’s *t* test (**A**) or 1-way ANOVA followed by Tukey’s multiple-comparisons test (**B** and **D**). *P < 0.05 and **P < 0.01 versus sham or ERRγ only; ^#^*P* < 0.05 and ^##^*P* < 0.01 versus WT BDL, Ad-LacZ, or ERRγ+p300.

To determine the primary molecular target of Sirt6, we assessed the expression of genes involved in BA synthesis and metabolism. *Cyp7a1* transcript levels were increased by BDL in Sirt6-KO mice to a much greater extent than in WT mice (Supplemental Figure 6). Among different nuclear receptors known to regulate CYP7A1, *Nrih4* (FXR) and *Nr0b2* (SHP), negative modulators of *Cyp7a1*, were increased by BDL, whereas *Esrrg* (ERRγ) expression was suppressed by BDL. Consistent with changes in the mRNA level of *Cyp7a1*, CYP7A1 protein level was increased by BDL (Figure 4A). Interestingly, protein level of ERRγ was also increased in both genotypes with significantly higher expression observed in KO mice than WT mice, suggesting possible regulation of *Cyp7a1* transcription by ERRγ. More importantly, lysine acetylation of ERRγ was detected in the livers of mice that underwent BDL and was much greater in KO mice than WT mice (Figure 4A). Adenoviral reexpression of Sirt6, but not mSirt6, clearly decreased acetylated ERRγ and total ERRγ levels and also those of CYP7A1 (Figure 4B), indicating that Sirt6-mediated deacetylation of ERRγ might be coupled with protein destabilization and CYP7A1 repression. To investigate this further, we evaluated ERRγ deacetylation by Sirt6 in HEK293T cells after transfection of the cells with Sirt6 or mSirt6 together with p300 and ERRγ. Coimmunoprecipitation assays showed a direct physical interaction between ERRγ and Sirt6 and deacetylation of ERRγ by Sirt6 (Figure 4C). On the contrary, Sirt1 overexpression did not affect the acetylation of ERRγ (Supplemental Figure 7). ERRγ is a constitutive transcription factor that binds to estrogen-related response elements (ERREs) in the promoter or enhancer regions of its target genes (21, 22). Therefore, we next determined whether the ability of ERRγ to stimulate ERRE-dependent luciferase reporter gene was affected by p300 acetyltransferase or Sirt6 deacetylase. HEK293T cells were transfected with separate expression vectors for ERRγ and p300, along with an ERRE–firefly luciferase reporter construct. ERRγ was able to stimulate transcription of the luciferase construct, which was further increased by p300 but decreased by Sirt6 (Figure 4D). Overall, these results demonstrate that Sirt6 physically binds to and deacetylates ERRγ, thereby inhibiting its transcriptional activity.

**Figure 5:**
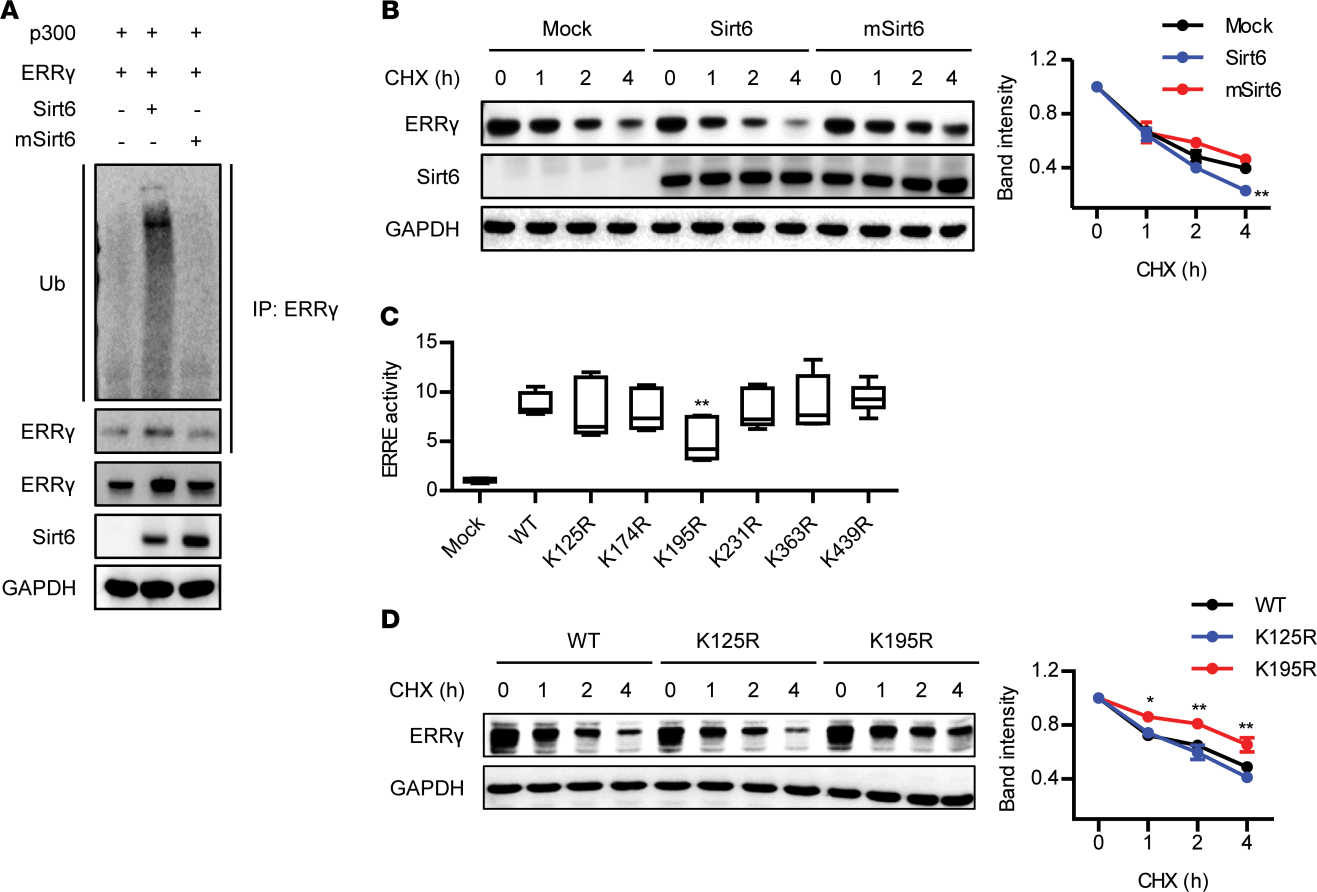
Regulation of ERRγ protein stability by Sirt6. (**A**) After transfection of HEK293T cells with the indicated plasmids in the presence of MG132 (2 μM), protein lysates were immunoprecipitated with anti-ERRγ antibodies and immunoblotted with anti-ubiquitin antibodies. (**B**) Cells transfected with p300 and ERRγ with or without either Sirt6 or mutant Sirt6 were treated with cycloheximide (CHX, 10 μg/mL) for the indicated times, and ERRγ protein levels were compared (*n* = 3). (**C**) Cells were transfected with p300 with WT or mutant ERRγ, and ERRE-luciferase activity was assayed (*n* = 4). (**D**) Cells were transfected with WT or mutant ERRγ (K125R and K195R) and then treated with CHX (10 μg/mL) for the indicated time periods. Protein levels of ERRγ were compared (*n* = 3). Values are shown as mean ± SEM. Comparisons were made using 1-way ANOVA followed by Tukey’s multiple-comparisons test. *P < 0.05 and **P < 0.01 versus mock or WT.

Lysine (de)acetylation is a major target for proteasome-mediated protein degradation. We next examined whether ubiquitination of ERRγ is affected by Sirt6. Sirt6 induced a huge increase in ubiquitination of ERRγ, leading to accelerated degradation of ERRγ protein when measured by cycloheximide chase analysis (Figure 5, A and B). Through liquid chromatography tandem mass spectrometry analysis, we were able to identify 6 lysine residues of ERRγ that were acetylated: K125, K174, K195, K231, K363, and K439 (Supplemental Figure 8). To determine functionally relevant lysine residue(s) that are linked with the transcriptional activity of ERRγ, we substituted each lysine residue with an arginine residue and tested the ability of the mutant proteins to stimulate ERRE-reporter activity and their effects on protein stability. When we expressed either WT ERRγ or KR-mutated ERRγ, only K195R significantly decreased ERRE-luciferase activity compared with ERRE-luciferase activity of WT ERRγ (Figure 5C). ERRγ stability of the K195R mutant was greater than that of WT ERRγ (Figure 5D), indicating that deacetylation of Lys195 of ERRγ mediates its protein degradation.

### Elimination of ERRγ abolishes the detrimental effects of Sirt6 deficiency.

**Figure 6:**
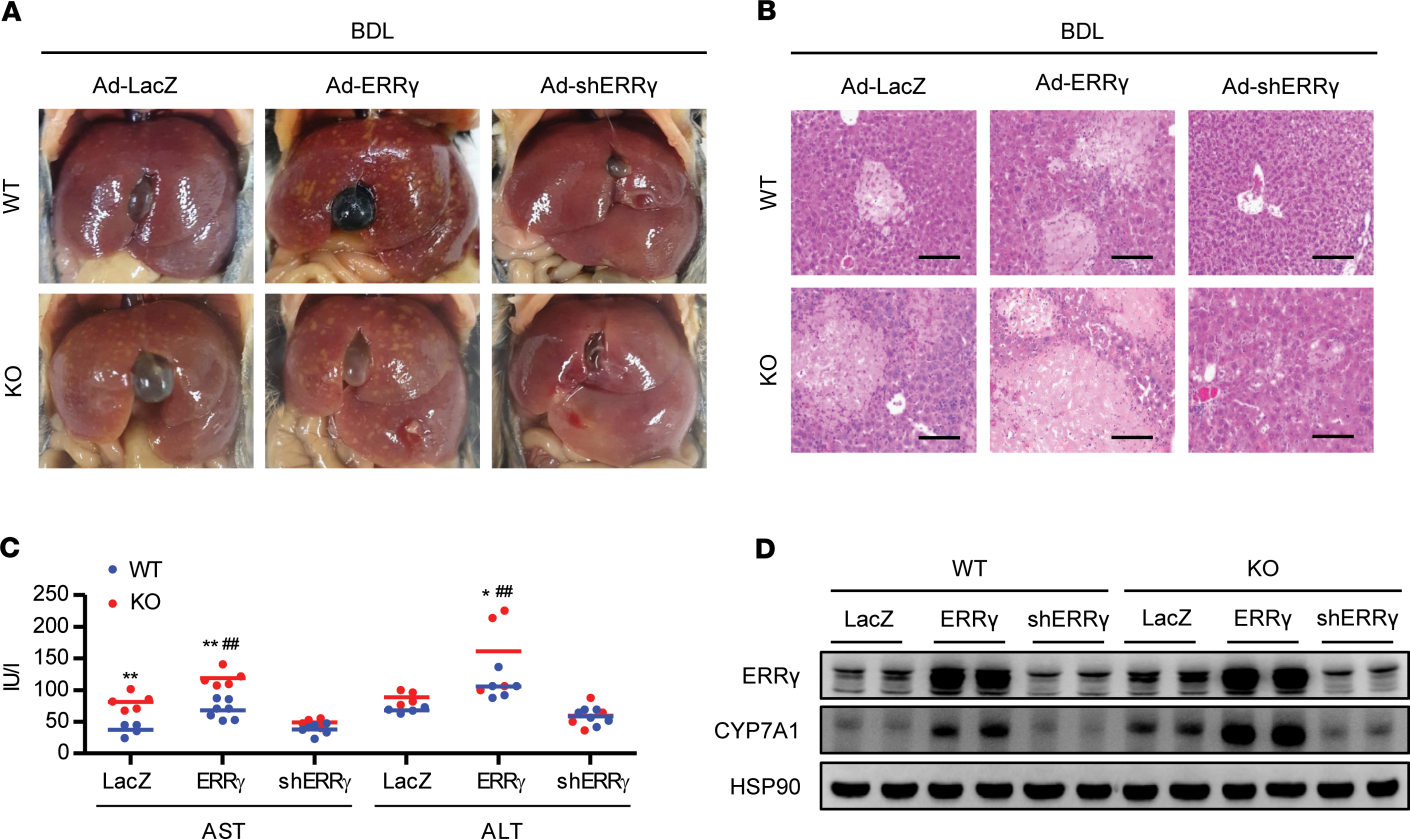
Ad-shERRγ abolishes the detrimental effects of Sirt6 deficiency. (**A**–**C**) WT and Sirt6-KO mice were injected intravenously with Ad-LacZ, Ad-ERRγ, or Ad-shERRγ and then subjected to BDL. Gross morphology of the liver, necrosis, and levels of AST and ALT (*n* = 4–6) were analyzed. Scale bar: 200 μm. (**D**) Analysis by Western blotting of ERRγ and CYP7A1. Values are shown as mean ± SEM. Comparisons were made using 1-way ANOVA followed by Tukey’s multiple-comparisons test. *P < 0.05 and **P < 0.01 versus WT; ^##^*P* < 0.01 versus Ad-LacZ.

We next tested if ERRγ contributed to the adverse effects of Sirt6 deficiency on cholestatic liver injury. Adenovirus-mediated overexpression of ERRγ in the liver of WT mice elicited a slight increase in liver/body weight ratio (Supplemental Figure 9A) and aggravated liver damage by BDL (Figure 6, A–C, and Supplemental Figure 9B). The injurious effects of ERRγ overexpression were much greater in Sirt6-KO mice than WT mice. Conversely, injection of Sirt6-KO mice with Ad-shERRγ completely abolished BDL-induced liver injury, leading to almost no difference between WT and KO mice (Figure 6, A–C, and Supplemental Figure 9B). Of note, CYP7A1 expression correlated well with the level of ERRγ, indicating that ERRγ is a bona fide transcription factor for *Cyp7a1* (Figure 6D). Overall, these results indicate a potential role for ERRγ in cholestatic liver injury and demonstrate that it aggravates the effect of Sirt6 deficiency.

To assess whether acetylation of ERRγ plays a role in cholestasis-induced liver injury, we injected adenoviruses carrying either WT ERRγ or K195R-ERRγ into C57BL/6 mice and performed BDL. As shown in Supplemental Figure 10, histological and biochemical parameters relating to cholestatic liver injury were aggravated to a less extent in mice injected with Ad-K195R-ERRγ compared with those injected with Ad-WT-ERRγ. To be consistent with this, CYP7A1 expression in liver was markedly elevated in mice with Ad-ERRγ compared with mice with Ad-LacZ but was not elevated in mice with Ad-K195R-ERRγ. Together, these results indicate that ERRγ acetylation at Lys195 is critical for protein stability and cholestatic liver injury.

### Treatment of a small-molecule activator of Sirt6 prevents BDL-induced liver injury.

**Figure 7:**
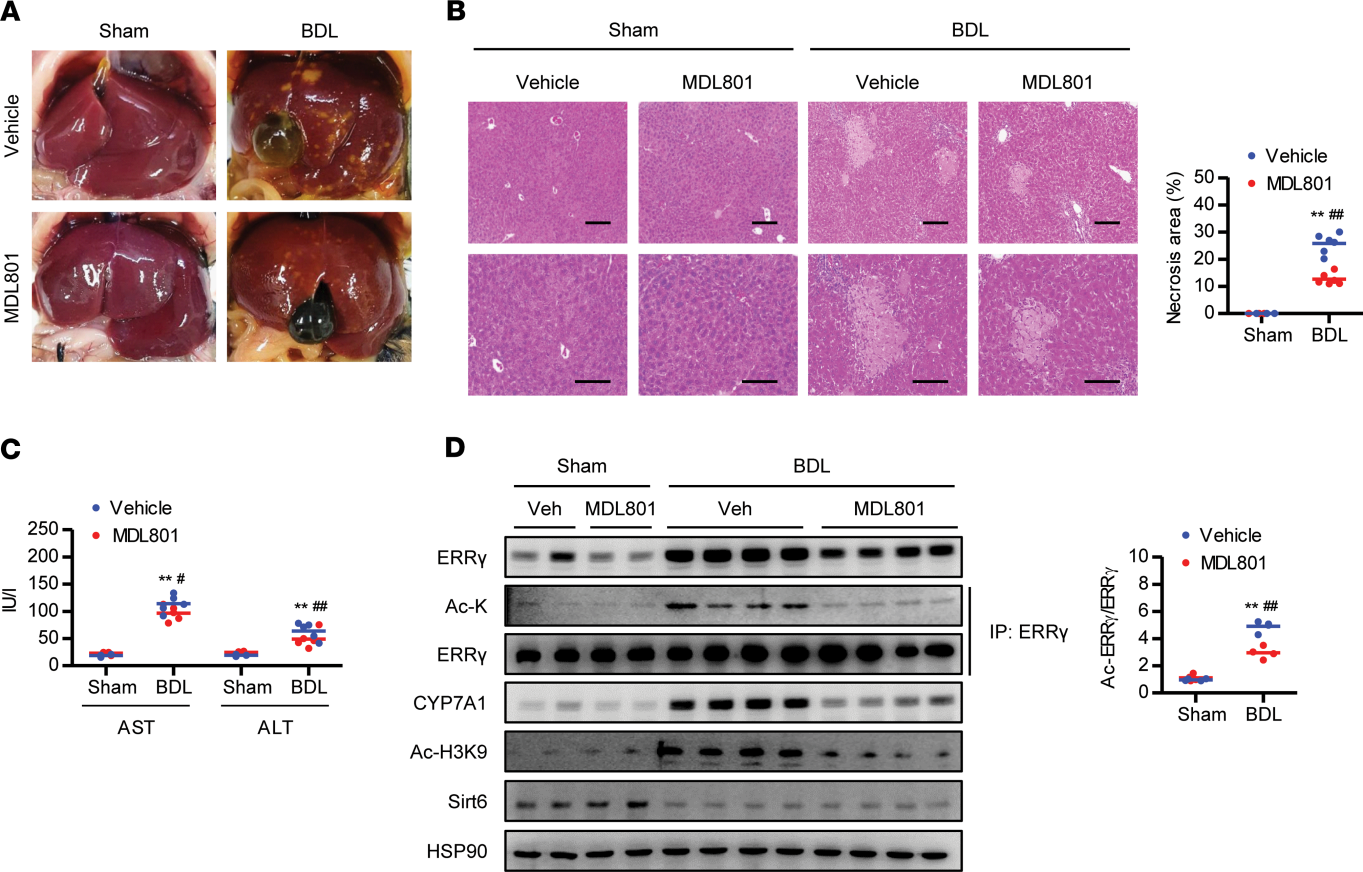
Prevention of BDL-induced liver injury by a small-molecule activator of Sirt6. C57BL/6 mice were treated with MDL801 (100 mg/kg) via intraperitoneal injection 1 day before BDL surgery and every other day after BDL for 5 times. Gross morphology of the liver (**A**), liver necrosis based on H&E staining (**B**), and serum levels of AST and ALT (**C**, *n* = 4–6) were analyzed. Scale bar: 200 μm. (**D**) Analysis by Western blotting of ERRγ and CYP7A1 (*n* = 4). Values are shown as mean ± SEM. Comparisons were made using paired 2-tailed Student’s *t* test (**B** and **D**) or 1-way ANOVA followed by Tukey’s multiple-comparisons test (**C**). **P < 0.01 versus sham; ^#^*P* < 0.05 and ^##^*P* < 0.01 versus vehicle.

Having elucidated the role of Sirt6 in protecting against BDL-induced liver injury, we examined whether activation of Sirt6 deacetylase could improve liver injury. To do so, we employed MDL801, which is a recently identified Sirt6 allosteric activator (23). Mice received MDL801 or vehicle via intraperitoneal injection. Gross morphology of the liver was improved by MDL801, but the liver/body weight ratio did not change (Figure 7A and Supplemental Figure 11A). MDL801 administration effectively suppressed BDL-induced liver injury based on histological and biochemical analyses (Figure 7, B and C, and Supplemental Figure 11B). The BDL-induced increase in the levels of total ERRγ and acetylated ERRγ and CYP7A1 in the liver were significantly suppressed by MDL801 treatment, confirming a Sirt6-ERRγ-CYP7A1 axis (Figure 7D).

### Hepatic expression of Sirt6 is inversely correlated with ERRγ in patients with cholestasis.

**Figure 8:**
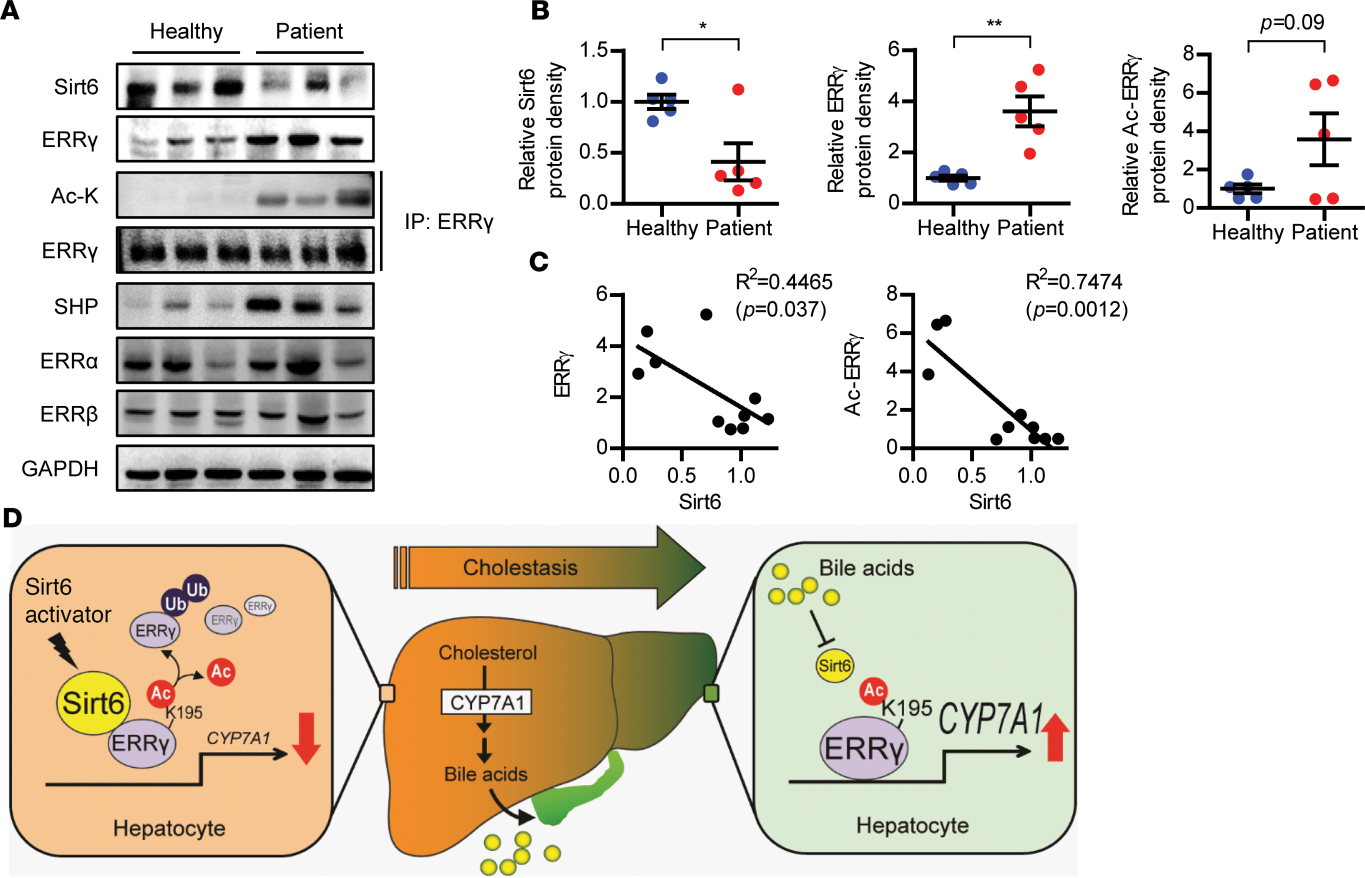
Increase in acetylated ERRγ in patients with cholestasis. (**A**) Expression levels of Sirt6 and total ERRγ and acetylated ERRγ were compared in liver tissues from healthy subjects and in segments of obstructed liver from patients with cholestasis. (**B**) Band intensities were quantified by densitometry (*n* = 5). (**C**) The coefficient of determination was used to evaluate the association between Sirt6 expression and that of either total ERRγ or acetylated ERRγ (*n* = 10). (**D**) Proposed summary. Comparisons were made using paired 2-tailed Student’s *t* test (**B**) or linear regression analysis (**C**). *P < 0.05 and **P < 0.01 versus healthy. ERRγ, estrogen-related receptor γ.

In an effort to demonstrate clinical relevance, we analyzed the expression of Sirt6 and ERRγ in liver samples from patients with cholestasis. These patients were specifically diagnosed with intrahepatic cholestasis due to ductal stone or cholangiocarcinoma, based on radiology, gross finding at surgery, and histology (data not shown). Although cholestatic injury was pronounced in obstructive legion, biochemical markers of cholestasis such as alkaline phosphatase (ALP) and gammaglutamyl trasferase (GGT) were only mildly elevated (Supplemental Table 1. Hepatic expression of ERRγ and its target genes and ERRγ acetylation were noticeably increased in patients with cholestasis (Figure 8, A and B, and Supplemental Figure 12). Conversely, and consistent with the animal data, Sirt6 expression was decreased in patients with cholestasis. Further analysis showed that hepatic ERRγ and its acetylation level were negatively correlated with Sirt6 protein level (Figure 8C). These findings suggest that the elevation in ERRγ expression due to Sirt6 downregulation in cholestatic liver contributes to the development and progression of this liver disease.

## Discussion

We demonstrated a potentially novel role for Sirt6 deacetylase in protecting against cholestatic liver injury and fibrosis and elucidated the underlying molecular mechanism. Sirt6 deacetylates and destabilizes ERRγ, thus inhibiting *Cyp7a1* transcription (Figure 8D). Hepatocyte exposure to BA represses Sirt6 expression, thus activating the ERRγ/CYP7A1 pathway and aggravating cholestatic liver injury in mice. A negative association between the expression of Sirt6 and ERRγ or its acetylation was also demonstrated in liver samples from patients with cholestasis. More importantly, we showed that Sirt6 activation by a small-molecule activator ameliorated cholestatic liver injury in BDL mice. This study provides proof of concept that Sirt6 activators may be therapeutic agents for cholestatic liver injury and fibrosis.

Reducing BA synthesis is one of the main strategies in the treatment of cholestasis. In this regard, elucidating the underlying molecular mechanism of CYP7A1 regulation is important. BA activation of hepatic FXR induces SHP, which inhibits liver receptor homolog 1, a monomeric orphan receptor necessary for *Cyp7a1* expression, thereby providing negative feedback repression of *Cyp7a1* (5). The currently available drug, obeticholic acid, activates the FXR/SHP pathway, thus repressing BA synthesis (4). Interestingly, both CYP7A1 and SHP expression were increased by BDL in our study, which was unexpected but consistent with previous findings (24, 25), suggesting the presence of other critical mediators of CYP7A1 induction. Moreover, we observed that Sirt6 overexpression reduced both SHP and CYP7A1 expression, indicating that the repressive effect of Sirt6 on CYP7A1 is independent of FXR/SHP signaling. Consistent with a previous report that demonstrated that ERRγ binds to the *Cyp7a1* gene promoter and increases BA synthesis (7), we observed a simultaneous increase in ERRγ and CYP7A1 levels in the livers of BDL mice. Because ERRγ and Sirt6 mRNA levels were decreased by BDL, we speculated that Sirt6 deacetylase might negatively regulate ERRγ protein stability. As such, identification of Sirt6 as a molecule to inhibit CYP7A1, which decreases protein level of ERRγ, can expand our understanding of BA synthesis and cholestasis.

ERR family members regulate genes involved in mitochondrial function and energy homeostasis. In the present study, only expression of ERRγ was increased by BDL, whereas expression of ERRα and ERRβ remained unchanged, confirming ERRγ-specific induction of CYP7A1. Transcriptional activity of ERRγ varies based on the presence or absence of coregulators and posttranslational modifications, but whether ERRγ is acetylated has not been studied previously to our knowledge. Based on the initial finding that ERRγ coimmunoprecipitates Sirt6, we investigated the possibility of direct deacetylation of ERRγ by Sirt6 and the biological relevance of this in cholestasis. We found that p300 overexpression was able to induce an increase in the acetylation and transactivation of ERRγ, which was inhibited by Sirt6 overexpression but not mutant Sirt6 overexpression. We further analyzed potential deacetylation residues in ERRγ targeted by Sirt6. Of the 6 lysine sites of acetylation in ERRγ, only Lys195 was critical for both protein stability and transactivational activity of ERRγ.

It is worth noting that although the role of Sirt1 in FXR/BA metabolism has been studied intensively, its role and therapeutic implications in cholestasis are unclear. Sirt1 activation by SRT1720 is protective in mice with cholestasis induced by cholic acid feeding (26). However, Sirt1 must be fine-tuned, given that prolonged Sirt1 activation leads to FXR degradation and that overexpression of Sirt1 aggravates liver injury (27), whereas depletion of Sirt1 is moderately protective in BDL mice (28). Meanwhile, Sirt1 has been shown to inhibit the transactivation of ERRγ due to complex formation with the corepressor of ERRγ at the ERRE without direct interaction with ERRγ (29). The expression of Sirt1 in cholestasis varies in different studies; Sirt1 expression was unaltered in BDL mice in our study, whereas a previous study reported an increase in Sirt1 expression in liver samples from patients with cholestasis (27). Taken together, our results indicate that Sirt6 regulates ERRγ function, which has important therapeutic implications for treating obstructive cholestasis.

BDL induced a huge increase in liver inflammation and fibrosis, whereas Sirt6 activation markedly inhibited these responses. The antiinflammatory function of Sirt6 has been well established by us and others and is mediated by limiting NF-κB target gene promoters or by inhibiting other inflammatory signaling molecules (11, 30). The observed hepatoprotective effects of Sirt6 in BDL mice may also be ascribed to its antiinflammatory action in addition to CYP7A1 repression. Cholestasis-associated liver damage is initiated by hepatocytes that secrete proinflammatory cytokines/chemokines, thereby contributing to inflammation (1, 20). Various molecules such as CCL2, CXCL1, CXCL2, and ICAM-1 have been reported to be important in neutrophil trafficking in the liver, which explains why liver injury occurs during cholestasis (19, 20, 31). We observed that neutrophil infiltration as well as transcript levels of the aforementioned molecules in the liver were dramatically increased by BDL along with macrophage number and that this was more pronounced in Sirt6-KO mice than WT mice, confirming the ability of Sirt6 to inhibit chemokine expression and monocyte/macrophage migration (10, 11).

In summary, we showed that Sirt6 activation inhibits CYP7A1 expression, thereby protecting against cholestatic liver injury and fibrosis. We are optimistic that these findings will promote the development of allosteric activators of Sirt6 for treatment of cholestasis and associated liver disease. This work also provides a mechanism for ERRγ regulation and expands our understanding of the molecular mechanism of cholestasis.

## Methods

### Animals.

Hepatocyte-specific Sirt6-KO mice (*Sirt6*^fl/fl^ *albumin-Cre*) were generated as described previously (10). To induce cholestatic liver injury, we subjected male KO mice and WT littermates to BDL surgery or fed mice a diet supplemented with 0.1% DDC (MilliporeSigma).

### Histology.

Paraffin sections of liver tissue (5 μm) were stained with H&E to evaluate areas of necrosis. After deparaffinization, sections were immunostained with antibodies against F4/80 (ab6640), CK19 (ab52625, Abcam), or Gr-1 (LS-C112469, Lifespan Bioscience). Apoptosis in the liver was determined by TUNEL staining using a kit (Promega). Sirius red staining was performed on paraffin sections using saturated picric acid containing 0.1% DirectRed 80 (MilliporeSigma). Stained areas were quantified by iSolution DT 36 software (Carl Zeiss), and results were expressed as a percentage of area.

### Cell culture and transient transfection.

Primary hepatocytes and KCs were isolated by perfusing livers with collagenase type IV (MilliporeSigma) as described previously (32). Hepatocytes and KC cocultures were prepared by culturing primary hepatocytes at the bottom of 24-well cell culture plates and KCs on 8 μm Transwell membrane inserts (BD Life Sciences).

The human embryonic kidney cell line HEK293T was obtained from the American Type Culture Collection. Exogenous proteins were expressed by transfecting HEK293T cells with 0.1–1 μg of ERRγ, Sirt6, Sirt6-H133Y (mSirt6), and p300 using Lipofectamine 3000 (Invitrogen). For the ERRγ reporter gene assay, 1 μg of a plasmid containing a promoter with an ERRE-driving luciferase expression was used.

### Site-directed mutagenesis.

Acetylation mutants of ERRγ were generated using a site-directed mutagenesis kit (Agilent Technologies) by converting each lysine residue (K125, K174, K195, K231, K363, and K439) to arginine (codon change from AAG or AAA to AGG or AGA). Mutations were confirmed by DNA sequencing performed at SolGent Co., Ltd.

### Antibodies.

Antibodies were used against the following proteins: Sirt1 (ab50517), Sirt5 (ab195436), ERRα (ab76228), ERRβ (ab19331), α-SMA (ab5694), collagen 1A1 (ab34710, Abcam); Sirt2 (sc-28298), SHP (sc-271511), and CYP7A1 (sc-518007, Santa Cruz Biotechnology); Sirt4 (3224) and Sirt7 (3099, Biovision); Sirt3 (5490s), Sirt6 (12486s), Ac-lysine (Ac-K) (9441s), p-STAT3 (Y705) (9145s), STAT3 (12640s), Bax (2772s), cleaved caspase-3 (9664s), p-ERK (4376s), ERK (4695s), p-JNK (4671s), JNK (9258s), p-p38 (9211s), p38 (9212s), and p-JAK2 (4406s; Cell Signaling Technology); Bcl2 (BS1511) and GAPDH (AP0066, Bioworld Technology); ERRγ (PP-H6812-00, Perseus Proteomics); or HSP90 (ADI-SPA-836-F, Enzo Life Sciences).

### Additional methods.

More details of the methods used are provided in Supplemental Methods.

### Statistics.

Data were expressed as mean ± SEM. Statistical comparisons were made using 1-way ANOVA followed by Fisher’s post hoc analysis. The significance of differences between 2 groups was determined using the Student’s unpaired 2-tailed *t* test. A *P* value less than 0.05 was considered significant.

### Study approval.

All animal experiments were performed in accordance with the *Guide for the Care and Use of Laboratory Animals* (National Academies Press, 2011). The study protocol of animal experiments was approved by the Institutional Animal Care and Use Committee of Chonbuk National University (approval CBNU: 2018-105). Human liver tissues were obtained from the Chonbuk National University Hospital Biobank with informed consent from the patients (Supplemental Table 1), and the study was approved by the Institutional Review Board of Chonbuk National University (approval CBNUH: 2019-04-057).

## Author contributions

LH, IHB, JW, YM, JDY, SYN, and JKS performed the experiments and analyzed the data. HSC, EJB and BHP designed the experiments, interpreted the data, and wrote the manuscript. All authors reviewed the manuscript.

## Supplementary Material

Supplemental data

## Version 1 07/23/2020

In-Press Preview

## Version 2 09/03/2020

Electronic publication
