# Supplementary material for: ERRγ suppression by Sirt6 alleviates cholestatic liver injury and fibrosis
Source: JCI Insight. 2020 Sep 3;5(17):e137566. doi: 10.1172/jci.insight.137566 (PMC7526444; doi:10.1172/jci.insight.137566)
Supplement: Supplemental data [file jciinsight-5-137566-s186.pdf]

ERRγ suppression by Sirt6 alleviates cholestatic liver injury and fibrosis

Lihua Hao[1*], In Hyuk Bang[1*], Jie Wang[1], Yuancheng Mao[1], Jae Do Yang[2], Soon-Young Na[3], Jeong Kon Seo[4], Hueng-Sik Choi[3], Eun Ju Bae[5¶], and Byung-Hyun Park[1¶]

Departments of [1]Biochemistry and [2]Surgery, Chonbuk National University Medical School, Jeonju, Jeonbuk 54896, Republic of Korea

[3]School of Biological Sciences and Technology, Chonnam National University, Gwangju 61186, Republic of Korea

[4]UNIST Central Research Facilities, UNIST, Ulsan 44919, Republic of Korea

[5]College of Pharmacy, Chonbuk National University, Jeonju, Jeonbuk 54896, Republic of Korea

[*]These authors contributed equally to this work

Contents

1. Supplementary methods

2. Supplementary tables

3. Supplementary figures

4. Supplementary references

1. Supplementary methods

Animals

Male Sirt6 KO mice and wild-type littermates were subjected to bile duct ligation at 8–10 weeks of age. After midline laparotomy, the common bile duct was exposed and ligated with 6–0 silk sutures twice. In the sham operation, the bile duct was touched and the abdomen was closed with 6-0 suture and mice were allowed to wake up on a heating pad. On day 3 after bile duct ligation, some mice were sacrificed to check neutrophil infiltration, and on day 10, serum was collected and the liver was harvested to isolate primary hepatocytes or processed for qPCR, western blotting, and histology. Adenoviruses were administered 2 days before BDL surgery. MDL801 (Chemscene, Monmouth Junction, NJ, USA) dissolved in 4% DMSO + 48% PBS + 48% PEG400 was administered to mice intraperitoneally at a dose of 100 mg/kg body weight 1 day before BDL surgery and every other day after BDL surgery (five times).

Preparation of recombinant adenovirus

Adenoviruses expressing Sirt6 (Ad-Sirt6), the catalytically inactive mutant Sirt6-H133Y (Ad-mSirt6), ERRγ (Ad-ERRγ), shERRγ (Ad-shERRγ), and β-galactosidase (Ad-LacZ) were prepared as described previously (1, 2). To prepare ERRγ-K195R-expressing adenovirus, mouse ERRγ-K195R cDNA was inserted into the pAdTrack-CMV vector (Addgene, Watertown, MA, USA). The pAdTrack-CMV-ERRγ-K195R plasmid was cloned into the pAdEasy-1 vector (Agilent, Santa Clara, CA, USA). The plasmid was linearized with Pac I and transfected into 293A cells using Lipofectamine 3000 (Invitrogen, Carlsbad, CA, USA).

Isolation of primary hepatocytes and Kupffer cells

Primary hepatocytes and Kupffer cells were isolated from 8- to 10-week-old male Sirt6 KO mice and wild type (WT) littermates. Anesthetized mice were intubated in the inferior vena cava, and the liver was perfused at a flow rate of 0.35 ml/min with calcium-free 10 mM HEPES buffer (pH 7.4) for 2 min, followed by perfusion with 15 ml of HEPES buffer containing 5 μg of collagenase IV (Sigma-Aldrich) and 5 mM calcium chloride. Hepatocytes were re-suspended in Medium199 supplemented with 10% FBS, 10 units/ml penicillin, 10 μg/ml streptomycin, and 10 nM dexamethasone mixed with 42% Percoll and then centrifuged for 5 min at 1,300 rpm to remove dead cells. Live hepatocytes were plated at 1×10^{6} cells/well in 6-well culture dishes. After 5 h, culture medium was changed.

To isolate Kupffer cells, hepatocytes were removed by centrifugation at 500 rpm for 3 min and washed 3 times. After the last wash, the supernatant was transferred to a new 50 ml tube and centrifuged for 7 min at 1,600 rpm. The cell pellet was then resuspended in 4 ml 17.5% OptiPrep (Sigma-Aldrich, St. Louis, MO, USA) and loaded carefully into the bottom of a new tube containing 2 ml 11.5% OptiPrep and 2 ml HBSS (Sigma-Aldrich). After centrifugation at 2,700 rpm for 17 min, the Kupffer cells-enriched layer was harvested from the 11.5% and 17.5% OptiPrep interphase. After washing with MACS buffer (Miltenyi Biotech, Paris, France) and blocking with anti-mouse CD16/32 (eBioscience, San Diego, CA, USA) for 20 min, cells were incubated with anti-mouse CD11b biotin (eBioscience) for 30 min at 4°C, then washed and incubated with anti-biotin microbeads (Miltenyi Biotech) for 20 min at 4°C. Cells were washed twice more using MACS buffer to remove excess microbeads. The supernatant was removed and the cell pellet was re-suspended in MACS buffer, and the cell suspension was placed in a magnetic column (Miltenyi Biotech) for separation. After washing with MACS buffer, the

column was removed from the magnetic field, and the fraction containing the CD11b positive cells was eluted with MACS buffer. Cells were centrifuged at 1,800 rpm for 5 min, and the pellet was then resuspended in DMEM supplemented with 10% FBS and antibiotics and cells were plated at a density of 5×10^{5} cells/well in a 12-well plate.

Biochemical analysis

Serum levels of alanine aminotransferase (ALT), aspartate aminotransferase (AST), total and conjugated bilirubin (Asan Pharm, Seoul, Korea), bile acid (BioVision, Milpitas, CA, USA), and alkaline phosphatase (ALP) activity (Abcam, Cambridge, UK) were analyzed using specific kits.

Western blots and co-immunoprecipitation

Cell lysates (10 μg) were separated using 7–14% SDS-PAGE and transferred to PVDF membranes. After blocking with 5% skim milk for 1 h, blots were probed with primary antibodies overnight at 4°C. For co-immunoprecipitations, 600 μg protein was incubated with anti-ERRγ, anti-Ac-Lys, or anti-Sirt6 antibodies overnight at 4°C followed by protein G-agarose for 2 h at 4°C. Blots were probed with primary antibodies against Ac-Lys, Sirt6, or ERRγ, and antibody signals were detected using a Las-4000 imager (GE Healthcare Life Science, Pittsburgh, PA, USA).

RNA isolation and qPCR

Total RNA was extracted from frozen liver tissues or primary hepatocytes using TRIzol reagent (Invitrogen). RNA was precipitated with isopropanol, dried using 70% ETOH, and dissolved in diethyl pyrocarbonate-treated distilled water. First-strand cDNA was generated using the random hexamer primer provided in the first-strand cDNA synthesis kit (Applied Biosystems, Foster City, CA, USA). Specific primers were designed using PrimerBank (https://pga.mgh.harvard.edu/primerbank, Table S2). qPCR reactions were performed in a final volume of 10 μl containing 10 ng reverse-transcribed total RNA, 200 nM forward and reverse primers, and PCR master mixture. qPCR was performed in 384-well plates using an ABI Prism 7900HT Sequence Detection System (Applied Biosystems).

Histology

Liver tissues were removed and placed immediately in fixative (10% formalin solution in 0.1 M PBS). Histological sections (5 μm) were cut from formalin-fixed paraffin-embedded tissue blocks. Tissue sections were stained with hematoxylin-eosin (H&E) under standard conditions. Immunohistochemical staining was performed using the DAKO Envision system (DAKO, Carpinteria, CA, USA). After deparaffinization and hydration, tissue sections were subjected to a microwave antigen-retrieval procedure in 0.01 M sodium citrate buffer. After blocking endogenous peroxidase, sections were incubated with protein blocking buffer to block nonspecific staining. Sections were incubated with primary antibodies against F4/80, Gr-1, and CK19 at 4°C overnight and secondary antibody for 30 min at room temperature. Peroxidase activity was detected with 3-amino-9-ethyl carbazole. The number of positive staining cells was counted in five microscopic fields (magnification, 100 or 200×) from each sample. TUNEL staining was performed following the manufacturer's instructions.

LC-MS/MS

After staining with colloidal Coomassie blue, protein gels were subjected to in-gel tryptic digestion as described by Shevchenko and co-workers (3). The resulting tryptic peptides were analyzed by LC-MS/MS. All mass analyses were performed on an LTQ-orbitrap mass spectrometer (Thermo Fisher Scientific, Bremen, Germany) equipped with a nanoelectrospray ion source. To separate the peptide mixture, we used a C_{18} reverse-phase HPLC column (150 mm × 75 μm ID) and an acetonitrile/0.1% formic acid gradient from 13 to 30% for 60 min at a flow rate of 300 nl/min. For MS/MS analysis, the precursor ion scan MS spectra (m/z 400~2,000) were acquired in the Orbitrap at a resolution of 60,000 at m/z 400 with an internal lock mass. The 20 most intensive ions were isolated and fragmented by collision-induced dissociation.

LC-MS/MS data processing for protein identification

All MS/MS samples were analyzed using the Sequest Sorcerer platform (Sagen-N Research, San Jose, CA, USA). Sequest was set up to search the mouse protein sequence database (51552 entries, UniProt, http://www.uniprot.org/), which includes frequently observed contaminants assuming digestion with trypsin. Sequest was searched with a fragment ion mass tolerance of 0.60 Da and a parent ion tolerance of 10.0 ppm. Carbamidomethyl of cysteine was specified in Sequest and X! Tandem as a fixed modification. Deamidation of asparagine and glutamine; methylation of glutamic acid, lysine, glutamine, and arginine; oxidation of methionine; acetylation of lysine, serine, and threonine, and the N-terminus; and

phosphorylation of serine, threonine, and tyrosine were specified in Sequest and X! Tandem as variable modifications. Additionally, Glu→pyro-Glu at the N-terminus, ammonia loss of the N-terminus, and Gln→pyro-Glu at the N-terminus were also specified in X! Tandem as variable modifications. Scaffold (Version 4.9.0, Proteome Software, Portland, OR, USA) was used to validate MS/MS-based peptide and protein identifications. Peptide identifications were accepted if they could be established at greater than 99.0% probability to achieve a false discovery rate less than 1.0% by the Scaffold Local FDR algorithm. Protein identifications were accepted if they could be established at greater than 99.0% probability to achieve an FDR less than 1.0% and contained at least two identified peptides. Protein probabilities were assigned by the Protein Prophet algorithm (4). Proteins that contained similar peptides and could not be differentiated based on MS/MS analysis alone were grouped to satisfy the principles of parsimony.

2. Supplementary tables

Table S1. Biochemical parameters of study subjects

Case	AST (IU/l)	ALT (IU/l)	TB (mg/dl)	CB (mg/dl)	ALP	GGT	Albumin	Metavir score#	Dx
Healthy									
1	42	24	1.09	0.25	63	32	3.7	NA	Healthy
2	24	37	0.62	0.2	68	37	4.6	NA	Healthy
3	26	22	0.99	0.32	70	42	4.4	NA	Healthy
4	29	38	0.72	0.21	87	75	4.6	NA	Healthy
5	26	27	0.69	0.23	58	15	4.7	NA	Healthy
Mean±SEM	29±4	29±4	0.8±0.1	0.2±0.0	69±6	40±11	4.4±0.2		
Patients									
6	30	15	0.55	0.15	88	19	4.3	F0	IHD St*
7	86	52	1.45	0.57	140	132	3.5	F0	IHD St
8	61	68	1.46	1.05	269	300	3.5	F0	IHD St*
9	52	74	0.9	0.26	59	100	3.4	F0	IHCC
10	22	18	0.69	0.24	74	12	4.4	F0	IHCC
Mean±SEM	50±12	45±13	1.0±0.2	0.5±0.2	126±43	113±58	3.8±0.2		
p value	0.12	0.28	0.41	0.27	0.21	0.21	0.08		

AST, aspartate aminotransferase; ALT, alanine aminotransferase; TB, total bilirubin; CB, conjugated bilirubin; ALP, alkaline phosphatase; GGT, γ-glutamyltransferase; Dx, diagnosis; NA, not applicable; IHD St, intrahepatic duct stone; IHCC, intrahepatic cholangiocarcinoma; *These patients underwent preoperative biliary drainage procedures (endoscopic retrograde cholangio-pancreatography for patient 6 and percutaneous transhepatic biliary drainage for patient 8) before segmental hepatectomy. #Metavir score was determined by histopathological evaluation in H&E stained liver sections (F0, no fibrosis; F1, portal fibrosis without septa; F2, portal fibrosis with few septa; F3, portal fibrosis with numerous septa; F4, cirrhosis).

Table S2. Sequences and accession numbers for primers (forward, FOR; reverse, REV) used in qPCR

Gene	Sequences for primers	Accession No.
Il1b	FOR:GGTCAAAGGTTTGGAAGCAG	NM_008361.4
	REV:TGTGAAATGCCACCTTTTGA	
Il6	FOR:CCACGGCCTTCCCTACTTC	NM_031168.2
	REV:TTGGGAGTGGTATCCTCTGTGA	
Tnfa	FOR:AGGGTCTGGGCCATAGAACT	NM_013693.3
	REV:CCACCACGCTCTTCTGTCTAC	
Nos2	FOR:TTCTGTGCTGTCCCAGTGAG	NM_010927.4
	REV:TGAAGAAAACCCCTTGTGCT	
Ccl2	FOR:ATTGGGATCATCTTGCTGGT	NM_011333.3
	REV:CCTGCTGTTCACAGTTGCC	
Ccr2	FOR:AGCACATGTGGTGAATCCAA	NM_009915.2
	REV:TGCCATCATAAAGGAGCCA	
Cxcl1	FOR:AATGAGCTGCGCTGTCAGTG	NM_008176.3
	REV:TGAGGGCAACACCTTCAAGC	
Cxcl2	FOR:ACCGACAACAGGAAGTGGAG	NM_009140.2
	REV:TGGACGTTTCACACAGTGGT	
Icam1	FOR:AACAGTTCACCTGCACGGAC	NM_010493.3
	REV:GTCACCGTTGTGATCCCTG	
Gapdh	FOR:CGTCCCGTAGACAAAATGGT	NM_008084.3
	REV:TTGATGGCAACAATCTCCAC	
Atca2	FOR:ACCAACTGGGACGACATGGAA	NM_007392.3
	REV:TGTCAGCAGTGTCGGATGCTC	
Colla1	FOR:TAGGCCATTGTGTATGCAGC	NM_007742.4
	REV:ACATGTTCAGCTTTGTGGACC	
Pdgfb	FOR:GAAGATCATCAAAGGAGCGG	NM_011057.4
	REV:CCTTCCTCTCTGCTGCTACC	
Tgfb1	FOR:GTGTGGAGCAACATGTGGAACTCTA	NM_011577.2
	REV:TTGGTTCAGCCACTGCCGTA	
Timp1	FOR:AGGTGGTCTCGTTGATTCGT	NM_011593.2
	REV:GTAAGGCCTGTAGCTGTGCC	
Esrrg	FOR:ACTTGGCTGACCGAGAGTTG	NM_011935.3
	REV:GCCAGGGACAGTGTGGAGAA	

Nrih4	FOR:CTTGATGTGCTACAAAAGCTGTG<br>REV:ACTCTCCAAGACATCAGCATCTC	NM_009108.2
Nr0b2	FOR:CGATCCTCTTCAACCCAGATG<br>REV:AGGGCTCCAAGACTTCACACA	NM_011850.3
Cyp7a1	FOR:AGCAACTAAACAACCTCCCAGTACTA<br>REV:GTCCGGATATTCAAGGATGCA	NM_007824.3
Tr4	FOR:GTCATGAGTCTCTCCACCATCCT<br>REV:GCTTTATCCGGTCACCAGAAA	NM_011630.3
Pparg	FOR:GCCTCGGGCTTCCACTAC<br>REV:AGATCCGATCGCACTTCTCA	NM_011145.3
Nr1i2	FOR:CAAGGCCAATGGCTACCA<br>REV:CGGGTGATCTCGCAGGTT	NM_010936.3
ACTB	FOR:GTCATGAGTCTCTCCACCATCCT<br>REV:GCTTTATCCGGTCACCAGAAA	NM_001101
FGG	FOR:GACGCTGCTACTTTGAAGTCC<br>REV:TGGATTTGCACCGTGTCTTTG	NM_021870
FGB	FOR:TTAGCCAGCTTACCAGGATGG<br>REV:TACCGGCTGTTCCTCTGTATT	NM_001184741
FGA	FOR:AGACATCAATCTGCCTGCAAA<br>REV:AGTGGTCAACGAATGAGAATCC	NM_000508
SIRT6	FOR:CCCACGGAGTCTGGACCAT<br>REV:CTCTGCCAGTTTGTCCCTG	NM_001193285

3. Supplementary figures

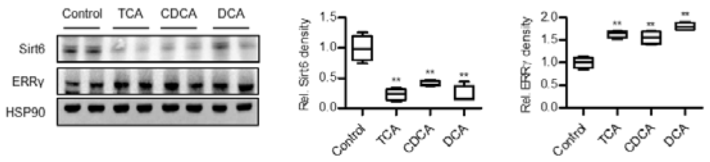

Figure S1. Expression of Sirt6 and ERRγ in hepatocytes. Primary hepatocytes were treated with 200 μM taurocholic acid (TCA), 125 μM chenodeoxycholic acid (CDCA), or 200 μM deoxycholic acid (DCA) for 6 h and protein levels of Sirt6 and ERRγ were analyzed (n=4). Values are means ± SEM. Comparisons were made using paired, two-tailed Student's *t* test. **, p<0.01 versus control.

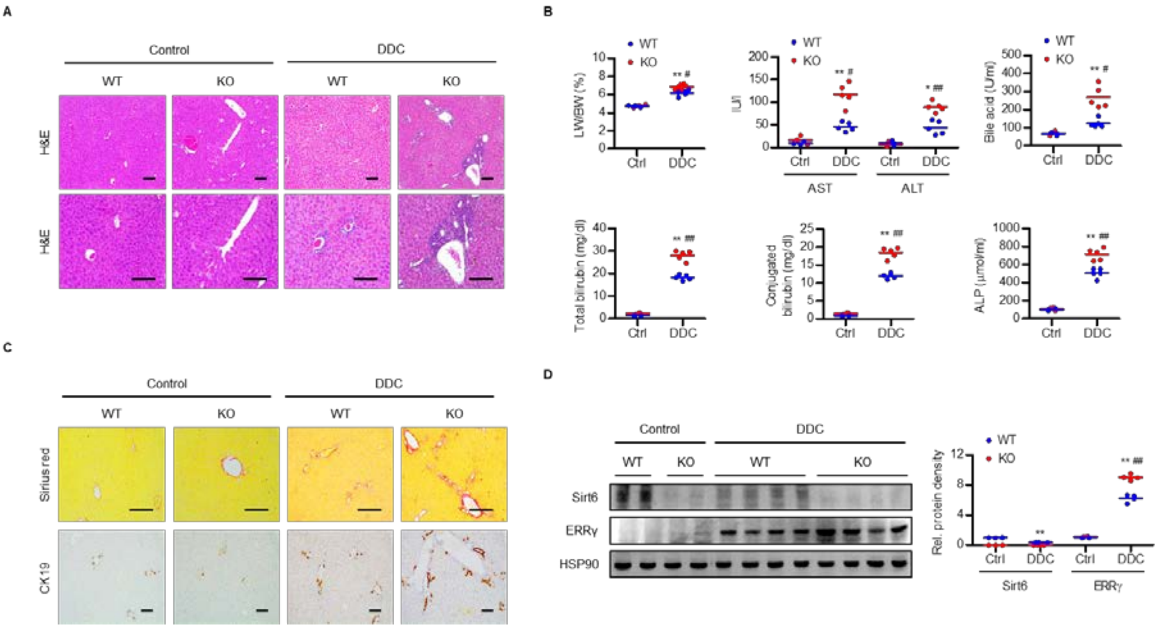

Figure S2. Aggravation of DDC diet-induced liver injury by Sirt6 deficiency. (**A**) WT and Sirt6 KO mice were fed the control or DDC diet for one week. Liver tissues were stained with H&E. Bars =200 μm. (**B**) Ratio of liver weight (LW) to body weight (BW) was determined, and serum levels of AST, ALT, bile acid, total bilirubin, conjugated bilirubin, and ALP were analyzed (n=4-6). (**C**) Sirius red staining and CK19 immunostaining in liver tissues (n=4). (**D**) Protein levels of Sirt6 and ERRγ were determined. Values are means ± SEM. Comparisons were made using paired, two-tailed Student's *t* test. *, $p<0.05$ and **, $p<0.01$ versus WT control; $^{\#}$, $p<0.05$ and $^{\#\#}$, $p<0.01$ versus WT DDC. Ctrl, control; DDC, 3.5-diethoxycarbonyl-1.4-dihydrocollidine.

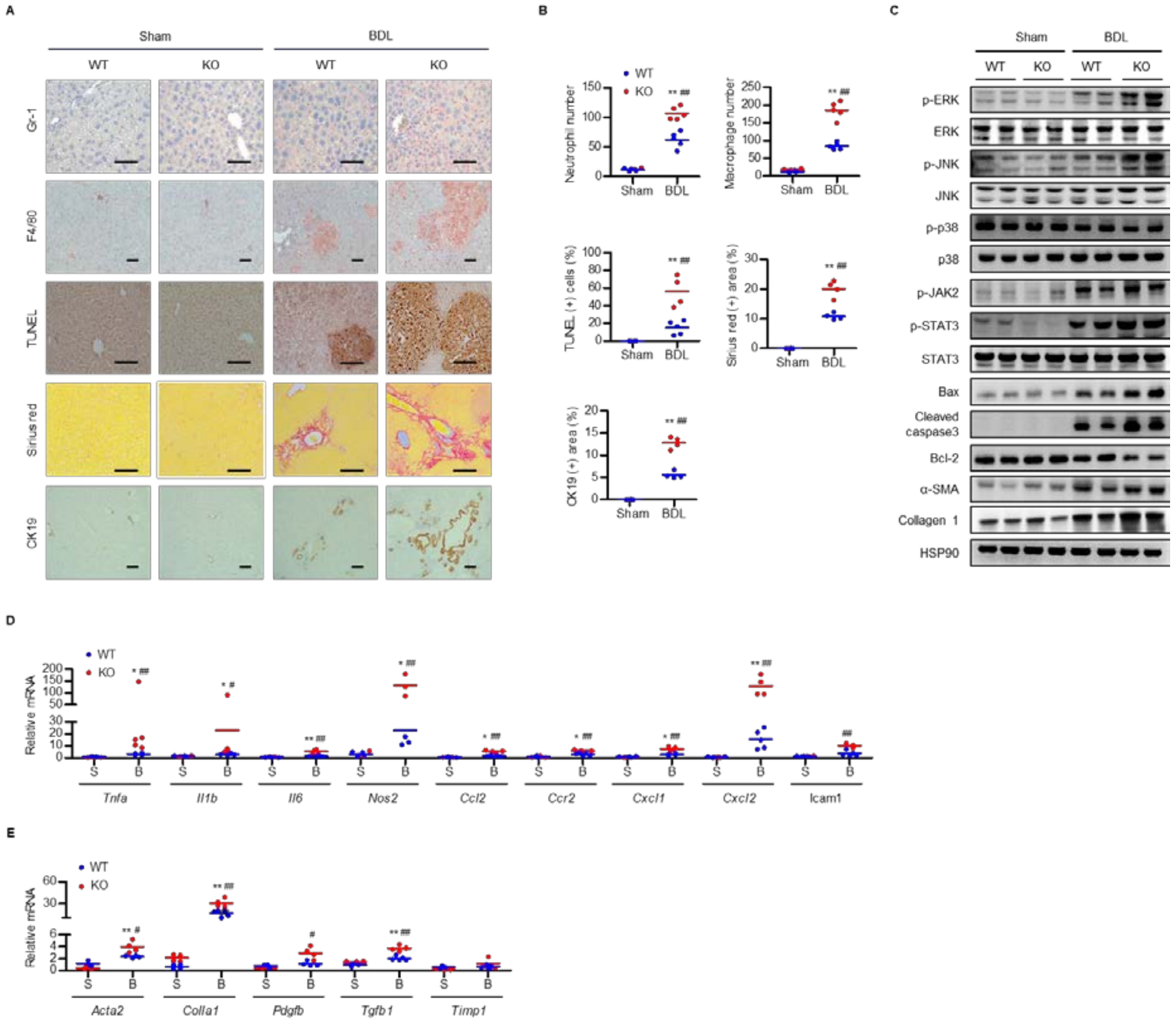

Figure S3. Worsening of liver inflammation, apoptosis, and fibrosis by Sirt6 deficiency. (**A**) Liver tissues retrieved 3 days (for Gr-1) or 10 days (F4/80, TUNEL, Sirius red, and CK19) after BDL were subjected to staining. Bars =200 μm. (**B**) Inflammation (Gr-1^{+} neutrophils and F4/80^{+} macrophages), apoptosis (TUNEL), pericellular fibrosis (Sirius red), and ductular reaction (CK19) were quantified (n=4-5). (**C-E**) After 10 days of BDL, proteins relating to cholestatic liver injury and mRNAs of genes related to inflammation and fibrosis were analyzed by western blotting and qPCR, respectively (n=3-5). Values are means ± SEM. Comparisons were made using paired, two-tailed Student's *t* test. *, p<0.05 and **, p<0.01 versus WT sham; $^{\#}$, p<0.05 and $^{\#\#}$, p<0.01 versus WT BDL. S, sham; B, BDL.

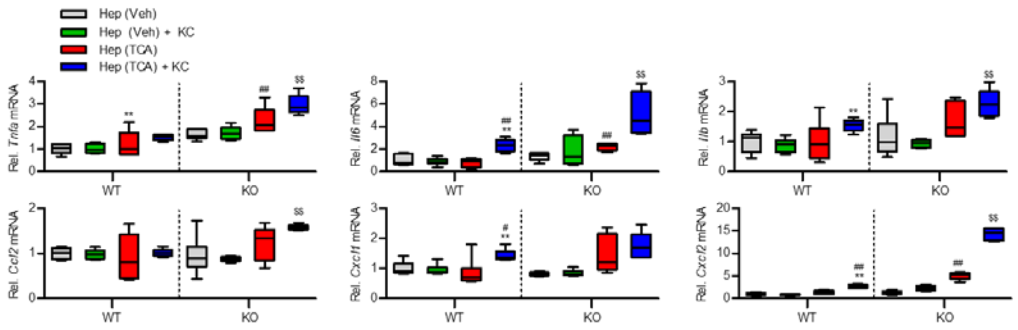

Figure S4. Enhancement of bile acid- and Kupffer cell (KC) co-culture-induced inflammation by Sirt6 deficiency. Primary hepatocytes (1×10^5) from WT or Sirt6 KO mice with or without Kupffer cell (1.5×10^5) co-culture were treated with 200 µM TCA for 12 h. mRNA levels of genes encoding cytokines and chemokines were analyzed by qPCR (n=6). Values are mean±SEM. Comparisons were made using one-way ANOVA followed by Tukey's multiple comparison test. **, *p*<0.01 versus WT Hep (Veh); $^{\#}$, *p*<0.05 and $^{\#\#}$, *p*<0.01 versus WT Hep (TCA); $^{\$\$}$, *p*<0.01 versus WT Hep (TCA)+KC. KC, Kupffer cell; Veh, vehicle; TCA, taurocholic acid.

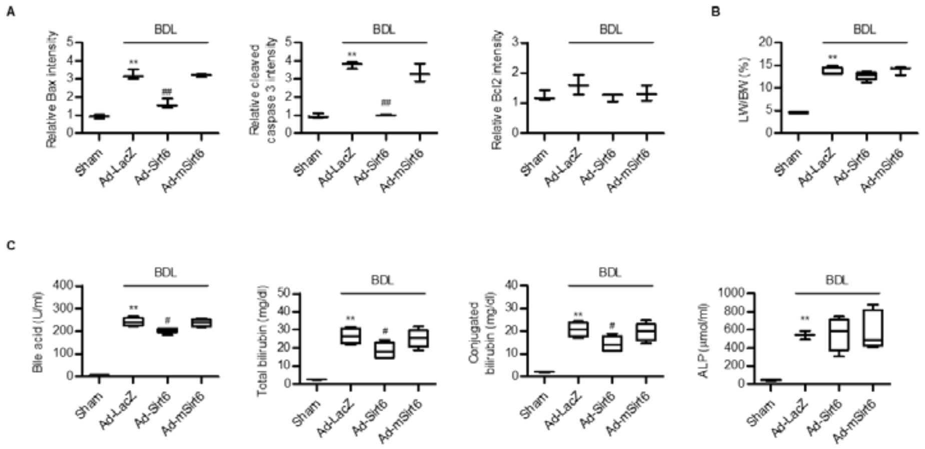

Figure S5. Attenuation of liver injury by re-expression of Sirt6 in BDL KO mice. All experiments were performed as described in the Fig. 3 legend. (**A**) Protein density of Figure 3D was measured (n=3). (**B**) The ratio of liver weight to body weight was analyzed (n=4). (**C**) Serum levels of bile acid, total bilirubin, conjugated bilirubin, and ALP were measured (n=5). Values are means ± SEM. Comparisons were made using one-way ANOVA followed by Tukey's multiple comparison test. **, p<0.01 versus sham; $^{\#}$, p<0.05 and $^{\#\#}$, p<0.01 versus Ad-LacZ.

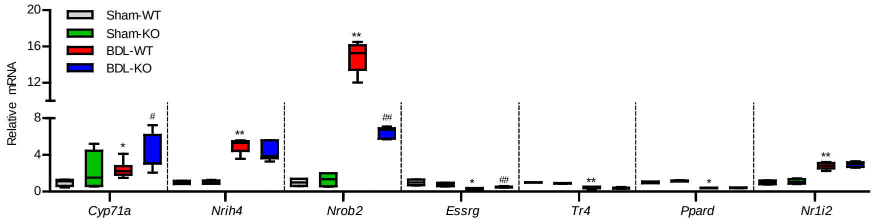

Figure S6. qPCR of *Cyp7a1* and nuclear receptors after BDL. After 10 days of BDL, mRNA levels of *Cyp7a1* and nuclear receptors were analyzed (n=4-7). Values are means ± SEM. Comparisons were made using one-way ANOVA followed by Tukey's multiple comparison test. *, p<0.05 and **, p<0.01 versus WT sham; $^{\#}$, p<0.05 and $^{\#\#}$, p<0.01 versus WT BDL.

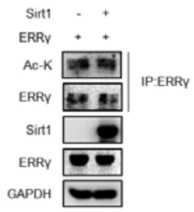

Figure S7. Deacetylation of ERRγ by Sirt1. After transfection of HEK293 cells with or without Sirt1, acetylation of ERRγ was determined.

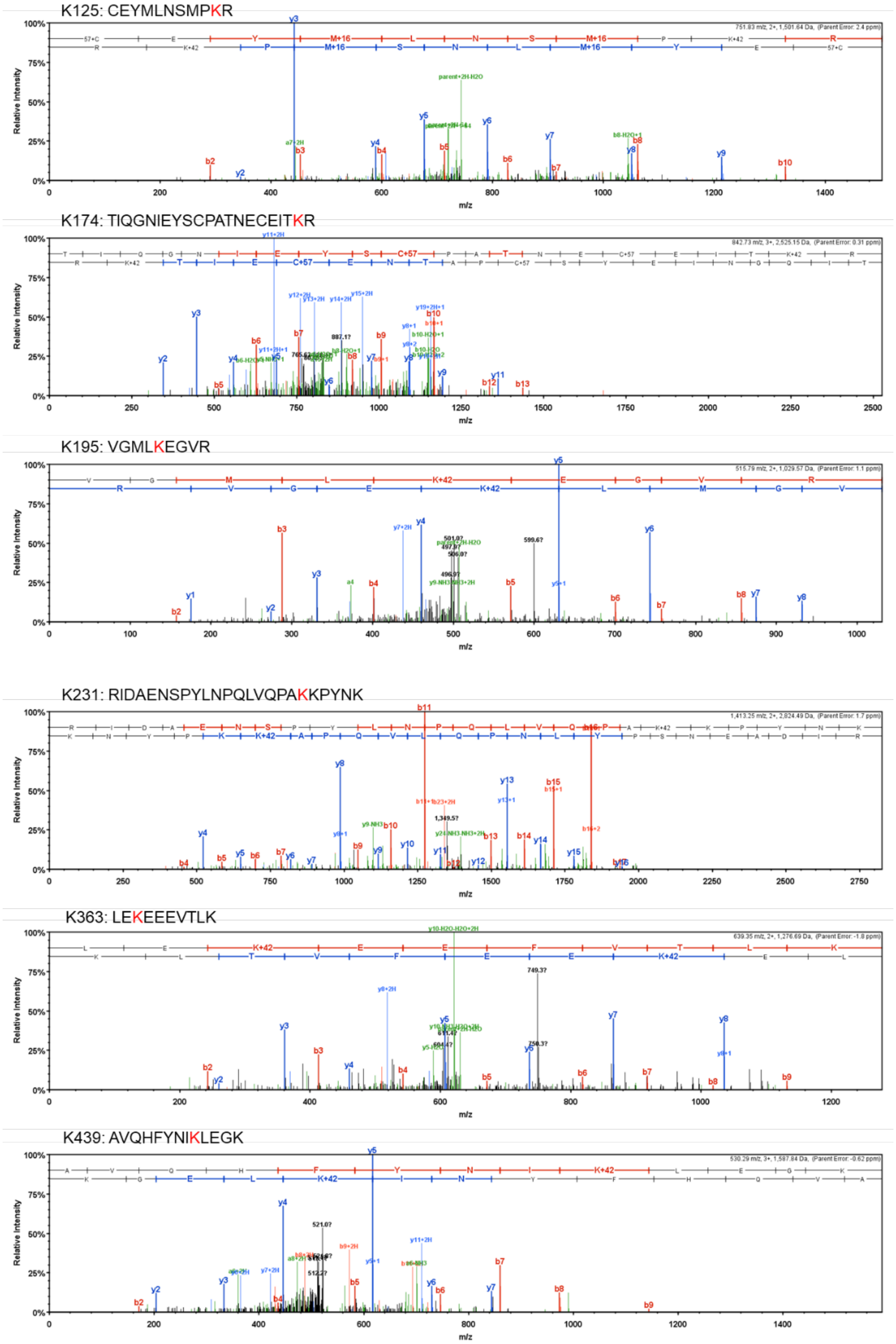

Figure S8. MS/MS spectra of ERRγ. HEK293T cells were transfected with either *Esrrg* or *p300*. Acetylation of ERRγ was determined using LC-MS/MS analysis after in-gel digestion. MS/MS spectra of acetylated ERRγ peptides containing K125, K174, K195, K231, K363, and K439 are shown.

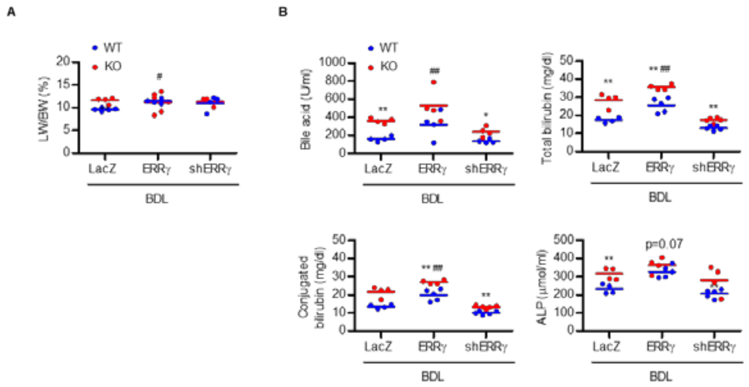

Figure S9. Ad-shERRγ abolishes the detrimental effects of Sirt6 deficiency. All experiments were performed as described in the Fig. 6 legend. (**A**) The ratio of liver weight to body weight was analyzed (n=4). (**B**) Serum levels of bile acid, total bilirubin, conjugated bilirubin, and ALP were measured (n=4). Values are means ± SEM. Comparisons were made using one-way ANOVA followed by Tukey's multiple comparison test. *, p<0.05 and **, p<0.01 versus WT; $^{\#}$, p<0.05 and $^{\#\#}$, p<0.01 versus Ad-LacZ.

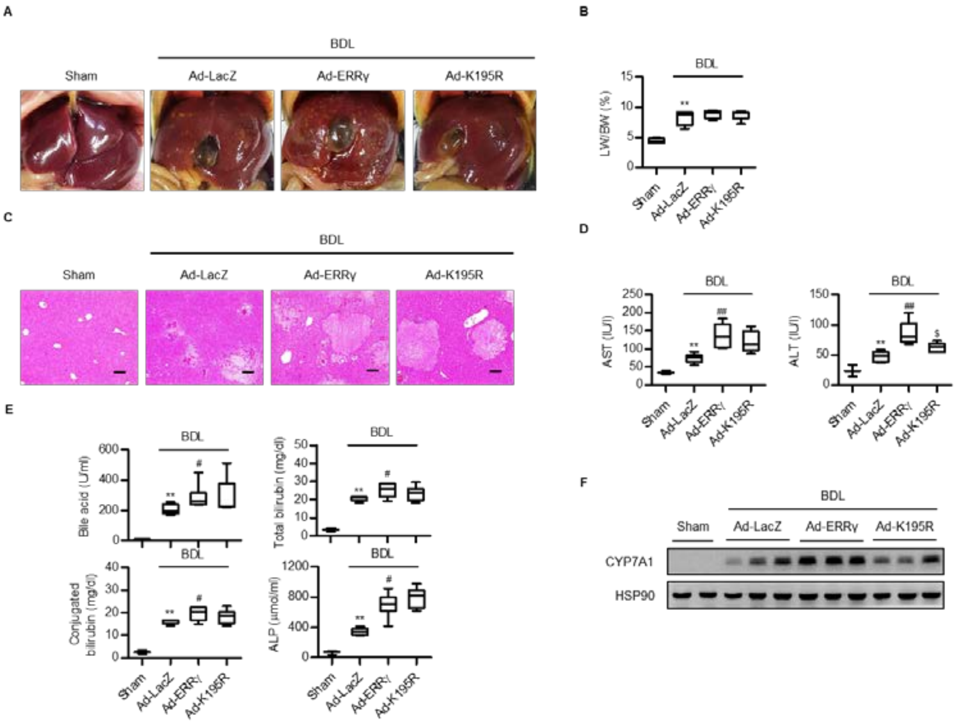

Figure S10. Abolishment of the detrimental effect of Sirt6 deficiency by Ad-K195R-ERRγ. C57BL/6 mice were injected intravenously with Ad-LacZ, Ad-ERRγ or Ad-K195R-ERRγ, followed by BDL. (**A**) Gross morphology of the liver. (**B**) The ratio of liver weight to body weight (n=4-6), (**C**) liver necrosis based on H&E staining, and (**D, E**) serum levels of AST, ALT, bile acid, total bilirubin, conjugated-bilirubin, and ALP (n=4-6) were analyzed. Bars =200 μm. (**F**) Western blot analysis of CYP7A1. Values are mean ± SEM. Comparisons were made using one-way ANOVA followed by Tukey's multiple comparison test. **, p<0.01 versus sham; $^{\#}$, p<0.05 and $^{\#\#}$, p<0.01 versus Ad-LacZ; $^{\$}$, p<0.05 versus Ad-ERRγ.

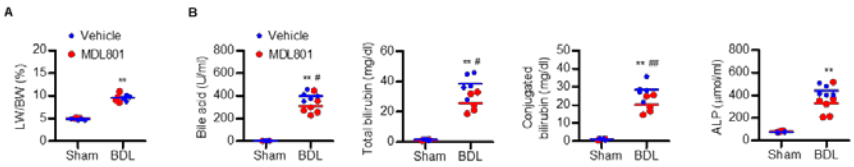

Figure S11. Prevention of BDL-induced liver injury by a small molecule activator of Sirt6. All experiments were performed as described in the Fig. 7 legend. (**A**) The ratio of liver weight to body weight was analyzed (n=4). (**B**) Serum levels of bile acid, total bilirubin, conjugated bilirubin, and ALP were measured (n=4). Values are means ± SEM. Comparisons were made using paired, two-tailed Student's *t* test. **, p<0.01 versus sham; $^{\#}$, p<0.05 and $^{\#\#}$, p<0.01 versus vehicle.

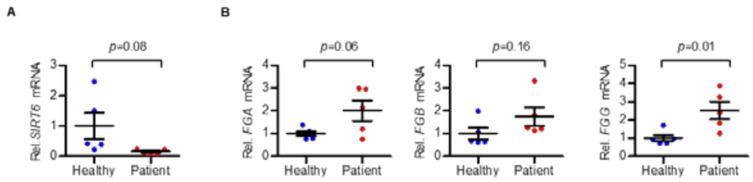

Figure S12. mRNA levels of SIRT6 and ERRγ target genes in patients with cholestasis. (**A, B**) mRNA levels of *SIRT6* and ERRγ target genes [fibrinogen alpha (*FGA*), fibrinogen beta (*GFB*), and fibrinogen gamma (*FGG*)] were compared in liver tissues from healthy subjects and patients with cholestasis. Values are mean ± SEM (n=5). Comparisons were made using paired, two-tailed Student's *t* test.

4. Supplementary references

1. Song MY, et al. Adipose sirtuin 6 drives macrophage polarization toward M2 through IL-4 production and maintains systemic insulin sensitivity in mice and humans. *Exp Mol Med* 2019;51:56.

2. Zhang Y, et al. Inverse agonist of ERRgamma reduces cannabinoid receptor type 1-mediated induction of fibrinogen synthesis in mice with a high-fat diet-intoxicated liver. *Arch Toxicol* 2018;92(9):2885-2896.

3. Shevchenko A, Tomas H, Havlis J, Olsen JV, Mann M. In-gel digestion for mass spectrometric characterization of proteins and proteomes. *Nat Protoc* 2006;1(6):2856-2860.

4. Nesvizhskii AI, Keller A, Kolker E, Aebersold R. A statistical model for identifying proteins by tandem mass spectrometry. *Anal Chem* 2003;75(17):4646-4658.
